# Protein kinase C is essential for viability of the rice blast fungus *M* *agnaporthe oryzae*

**DOI:** 10.1111/mmi.13132

**Published:** 2015-08-18

**Authors:** Tina J. Penn, Mark E. Wood, Darren M. Soanes, Michael Csukai, Andrew John Corran, Nicholas J. Talbot

**Affiliations:** ^1^ School of BiosciencesUniversity of ExeterGeoffrey Pope Building, Stocker RoadExeterEX4 4QDUK; ^2^ Biological SciencesSyngenta, Jeallott's Hill International Research CentreBracknellRG42 6EYUK

## Abstract

Protein kinase C constitutes a family of serine–threonine kinases found in all eukaryotes and implicated in a wide range of cellular functions, including regulation of cell growth, cellular differentiation and immunity. Here, we present three independent lines of evidence which indicate that protein kinase C is essential for viability of *M* *agnaporthe oryzae.* First, all attempts to generate a target deletion of *PKC* *1*, the single copy protein kinase C‐encoding gene, proved unsuccessful. Secondly, conditional gene silencing of *PKC* *1* by RNA interference led to severely reduced growth of the fungus, which was reversed by targeted deletion of the Dicer2‐encoding gene, *MDL* *2*. Finally, selective kinase inhibition of protein kinase C by targeted allelic replacement with an analogue‐sensitive *PKC* *1^AS^* allele led to specific loss of fungal viability in the presence of the PP1 inhibitor. Global transcriptional profiling following selective PKC inhibition identified significant changes in gene expression associated with cell wall re‐modelling, autophagy, signal transduction and secondary metabolism. When considered together, these results suggest protein kinase C is essential for growth and development of *M* *. oryzae* with extensive downstream targets in addition to the cell integrity pathway. Targeting protein kinase C signalling may therefore prove an effective means of controlling rice blast disease.

## Introduction

*Magnaporthe oryzae* is the causal agent of rice blast disease and is responsible for annual losses of up to 30% of the worldwide rice harvest (Talbot, 2003). Rice blast outbreaks occur throughout rice‐growing regions of the world and new strategies to control the disease are urgently required. In common with many cereal pathogens, *M. oryzae* uses a specialised infection structure called an appressorium to infect its host. Turgor in the appressorium is used to generate mechanical force to penetrate the rice leaf cuticle and allow subsequent invasive growth of the fungus in plant tissue. A number of signal transduction pathways are implicated in appressorium‐mediated plant infection and have been characterised as a potential means of developing new chemical intervention strategies for disease control (Rispail *et al*., 2009). Mitogen‐activated protein kinases (MAPKs), for instance, play a central role in appressorium morphogenesis (Xu, 2000) and three distinct MAPK signal transduction pathways have been identified in *M. oryzae* (Rispail *et al*., 2009). Appressorium development is regulated by the *PMK1* MAP kinase signalling cascade (Xu and Hamer, 1996) and *MPS1*, a component of the cell wall integrity pathway and functional homologue of yeast Mpk1, is necessary for appressoria to re‐polarise and breach the host cuticle (Xu *et al*., 1998). A third MAPK *OSM1*, meanwhile, regulates the cellular response to hyperosmotic stress (Dixon *et al*., 1999) and is a functional homologue of *S. cerevisiae HOG1*. These three MAP kinase pathways are well conserved in pathogenic fungi and associated with infection‐related development (Rispail *et al*., 2009). Conserved signalling pathways essential for the establishment of disease therefore offer potential targets for development of new anti‐fungal drugs, but the identification of key signalling pathways that are essential for fungal growth is arguably the best means by which highly effective drugs might be developed. In this study, we therefore set out to determine the role of the highly conserved protein kinase C in the rice blast fungus.

Protein kinase C (PKC) is found in all eukaryotes and is involved in activation and regulation of signal transduction pathways associated with growth, development and cell death. PKC belongs to a family of serine/threonine kinases and at least 10 isoforms have been identified in mammalian systems (Steinberg, 2008). PKC has been extensively studied in mammals, due in part to its association with a number of diseases ranging from cancer (Oka and Kikkawa, 2005) and diabetes (Lee *et al*., 1989) to Alzheimer's disease (Masliah *et al*., 1990; Khan *et al*., 2009). PKC has been implicated in regulation of a diverse range of cellular functions including cell proliferation (Oka and Kikkawa, 2005), cellular differentiation (Denning, 2004) and apoptosis (Martelli *et al*., 2004), but relatively little is known regarding its cellular substrates. Fungal protein kinase C enzymes are not well characterised, due in part to a failure to generate gene replacement mutants in filamentous fungi (Oeser, 1998; Franchi *et al*., 2005; Herrmann *et al*., 2006; Wang *et al*., 2011), but in the yeast *Saccharomyces cerevisiae*, *PKC1* is known to be involved in regulation of the cell integrity pathway (Heinisch *et al*., 1999) and the mutant is only viable under hyperosmotic conditions. In *Aspergillus nidulans*, temperature‐sensitive alleles of PkcA have been generated, suggesting a role for PKC in cell polarity, growth and development (Katayama *et al*., 2012) and recent evidence suggests an interplay with calcineurin‐mediated signalling and a role in the regulation of mitochondrial biogenesis, in addition to cell integrity (Colabardini *et al*., 2014). Although the function of PKC in filamentous fungi may be inferred from studies with *S*. *cerevisiae*, this model cannot be readily translated to the study of pathogenic development or invasive growth, characteristics associated with pathogens but not found in budding yeast.

In filamentous fungi, PKC is very likely to have more global effects on fungal physiology and development, and in this study, we attempted to establish its role in *M. oryzae*. We targeted the kinase using three different approaches: the conventional method of targeted gene replacement, downregulation of *PKC1* expression by RNA interference‐mediated gene silencing (Nakayashiki *et al*., 2005) and selective chemical inhibition of the kinase. We then performed transcriptional profiling, following specific PKC inhibition. When considered together, our results suggest that protein kinase C is essential for growth and development of the rice blast fungus and may play key roles in spore germination, cell wall biogenesis, polarised growth and hyphal development.

## Results

### Chemical inhibition of PKC activity prevented conidial germination and appressorium formation

**Figure 1:**
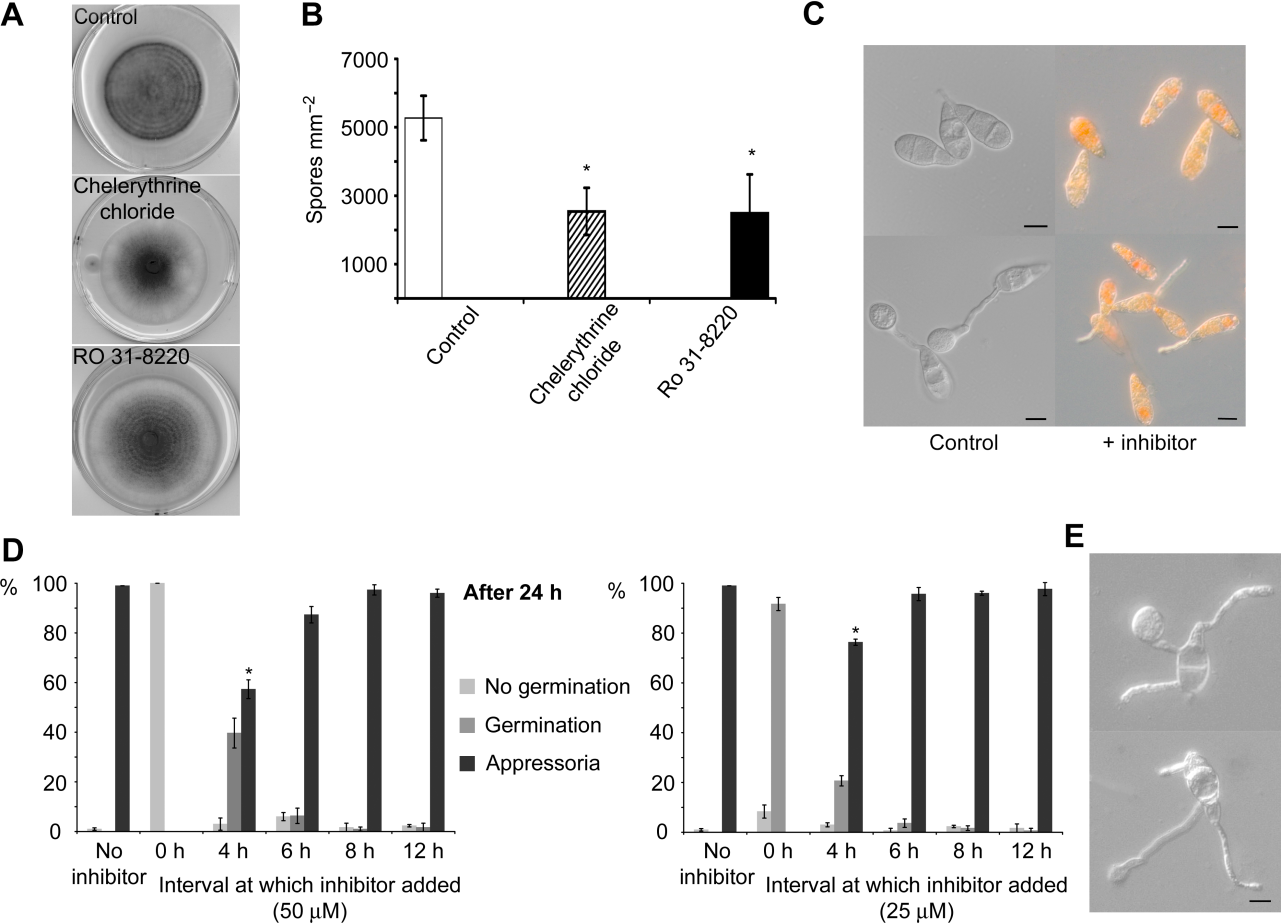
Pharmacological inhibition of *PKC* *1*. A. *M* *. oryzae* Guy11 cultured in the presence of known PKC inhibitors showed enhanced production of aerial hyphae, shown by white colony margins. B. Sporulation was significantly reduced in the presence of inhibitors (Student's t‐test *P < 0.05). C. The inhibitor Ro‐31–8220 was visible as an orange deposit within conidia and germ tubes. D. The frequency of conidial germination and appressorium development in the presence of chelerythrine chloride (left) and RO 31–8220 (right). Inhibitors were added to conidia incubated in water on plastic coverslips after 0, 4, 6, 8 or 12 h. After 24 h, the percentage that had elaborated a germ tube or formed an appressorium was recorded (*n* = 300). When inhibitors were added before 4 h, there was a significant inhibitory effect on appressorium development (*P < 0.05). E. When exposed to Ro 31–8220 (25 μM) at 0 h, 40% of conidia formed multiple germ tubes. Value shown is mean of three replications of the experiment. Error bar represents standard deviation for all bar charts.

To investigate the role of protein kinase C activity in *M. oryzae*, we first exposed the fungus to two known inhibitors of PKC, chelerythrine chloride (Herbert *et al*., 1990) and Ro‐31–8220, a staurosporine analogue (Beltman *et al*., 1996). The fungus was grown on complete medium agar supplemented with either inhibitor and in each case showed enhanced production of aerial hyphae (see white colony margins in Fig. 1A), but no significant effect on the rate of vegetative growth. The presence of either inhibitor did, however, result in significantly reduced levels of sporulation, *P* < 0.05 (Fig. 1B). The most striking effect of PKC inhibition was a reduction in conidial germination and appressorium formation. PKC inhibitors were added to conidial suspensions at varying concentrations and time intervals following incubation and observed after 24 h. Ro‐31–8220 was visible as an orange deposit within conidia and germ tubes, as shown in Fig. 1C. When added at 0 h at a concentration of 50 μM or higher, conidial germination was inhibited completely (Fig. 1D). After exposure to Ro 31–8220 at 25 μM concentration, 92 ± 3% of conidia germinated successfully, but there was no evidence of appressorium differentiation. Instead, 40% of conidia formed multiple germ tubes, as shown in Fig. 1E. Following exposure to 50 μM of Ro 31–8220 at 4 h, 97 ± 2% of conidia germinated but only 57 ± 5% formed appressoria, significantly fewer (*P* < 0.005) than in the absence of the inhibitor. Similarly, when 25 μM of Ro 31–8220 was added at 4 h, the frequency of appressorium formation was also significantly reduced (*P* < 0.05). The experiments were repeated by exposing *M. oryzae* conidia to chelerythrine chloride and the ability of conidia to elaborate appressoria was again severely affected (data not shown). There was no inhibitory effect from exposure to either inhibitor at any concentration when added after 6 h. Both the inhibitors employed in this study, while highly selective for PKC, may inhibit other kinases at high concentrations. However, as we carried out chemical inhibition of *PKC1* activity with two distinct inhibitors that are structurally unrelated, and the observed effects were identical, this strongly supports a role for PKC activity in conidial germination and appressorium formation of *M. oryzae*.

### A single PKC is encoded in the *M* *agnaporthe oryzae* genome

Interrogation of the *M. oryzae* genome sequence database http://www.broad.mit.edu/annotation/fungi/magnaporthe/) led to identification of a putative protein kinase C‐encoding gene, MG08689.5, with a coding region of 3895 bp interrupted by five introns, which we designated *PKC1*, and which is capable of encoding a 1183 amino acid protein. Preliminary analysis of the genome sequence (Dean *et al*., 2005) indicated that *M. oryzae PKC1* is a single copy gene and this was confirmed experimentally by restriction digest and Southern blot analysis (data not shown). While this is in contrast to mammalian systems, it is typical of fungal PKCs with the exception of the fission yeast, *Schizosaccharomyces pombe*, which has two copies of PKC‐encoding genes (Toda *et al*., 1993). PKC orthologues have been identified in several pathogenic fungi including *Cryptococcus neoformans* (Heung *et al*., 2004), *Colletotrichum trifolii* (Dickman *et al*., 2003), *Candidaalbicans* (Paravicini *et al*., 1996), *Cochliobolus heterostrophus* (Oeser, 1998), *Aspergillus niger* and *Trichoderma reesei* (Morawetz *et al*., 1996) and *Aspergillus nidulans* (Ichinomiya *et al*., 2007). Comparison of the amino acid sequence with known PKCs from other filamentous fungi revealed that the putative *PKC1* gene of *M. oryzae* contains all the conserved domains common to fungal PKCs (Fig. S1), including the characteristic PKC extended regulatory domain (Jacoby *et al*., 1997), and phylogenetically classified with known fungal PKCs (Fig. S2).

### *PKC* *1* is expressed during appressorium development and is cytoplasmically localised

**Figure 2:**
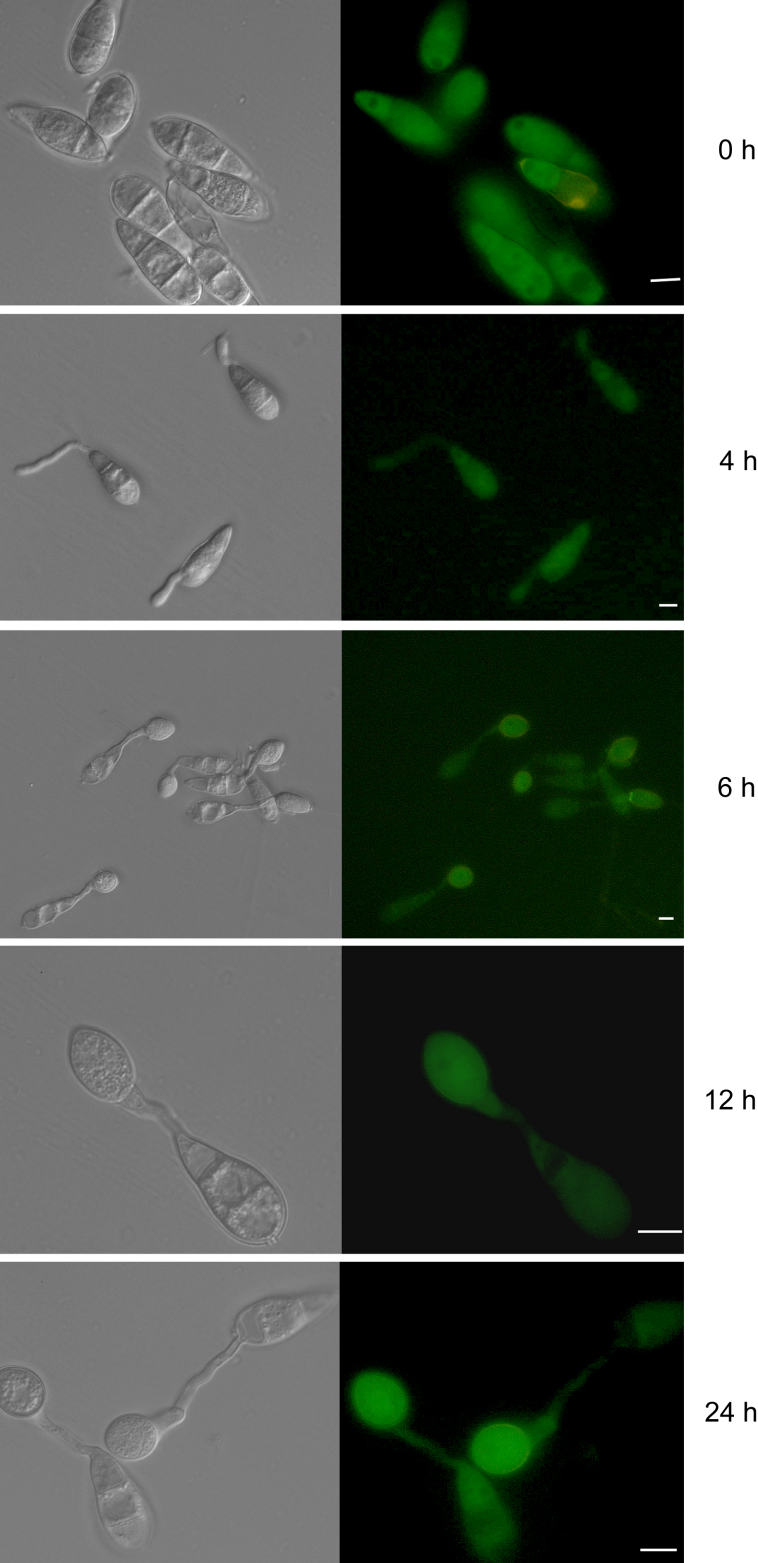
Expression of *M* *. oryzae* *PKC* *1* during conidial germination and appressorium development. A 5.9 kb genomic fragment containing the *PKC* *1* coding region and 2 kb of upstream promoter sequence were fused in‐frame to the s*GFP* green fluorescent protein‐encoding allele and introduced into *M* *. oryzae* wild type, Guy11. Conidia expressing *PKC* *1:* *GFP* were inoculated onto an unyielding surface (plastic coverslips) and germination and appressorium development were analysed by epifluorescence microscopy during 24 h. Scale bar = 10 μm.

We set out to identify the sub‐cellular localisation of PKC and the temporal and spatial pattern of *PKC1* expression. To determine the likely sub‐cellular location of PKC, we constructed and expressed a *PKC1:sGFP* gene fusion in *M. oryzae* strain Guy11. GFP fluorescence was consistently detected during conidial germination and appressorium development and predominantly located in the cytoplasm (Fig. 2). This is consistent with evidence from mammalian studies, which show that PKC resides in the cytoplasm and is translocated to its target upon activation. In *N. crassa*, PKC has been shown to localise to growing tips and the sub‐apical plasma membrane in actively growing hyphae and also in the septum at various stages of development (Khatun and Lakin‐Thomas, 2011). By contrast, no such specific localisation was observed in *M. oryzae* but relocation in *N. crassa* was transient in nature and the study employed an over‐expression promoter to enhance visualisation by microscopy (Khatun and Lakin‐Thomas, 2011), whereas we used the native *PKC1* promoter to drive expression of the *PKC1:sGFP* gene fusion.

### Protein Kinase C in *M* *. oryzae* is essential for viability

**Figure 3:**
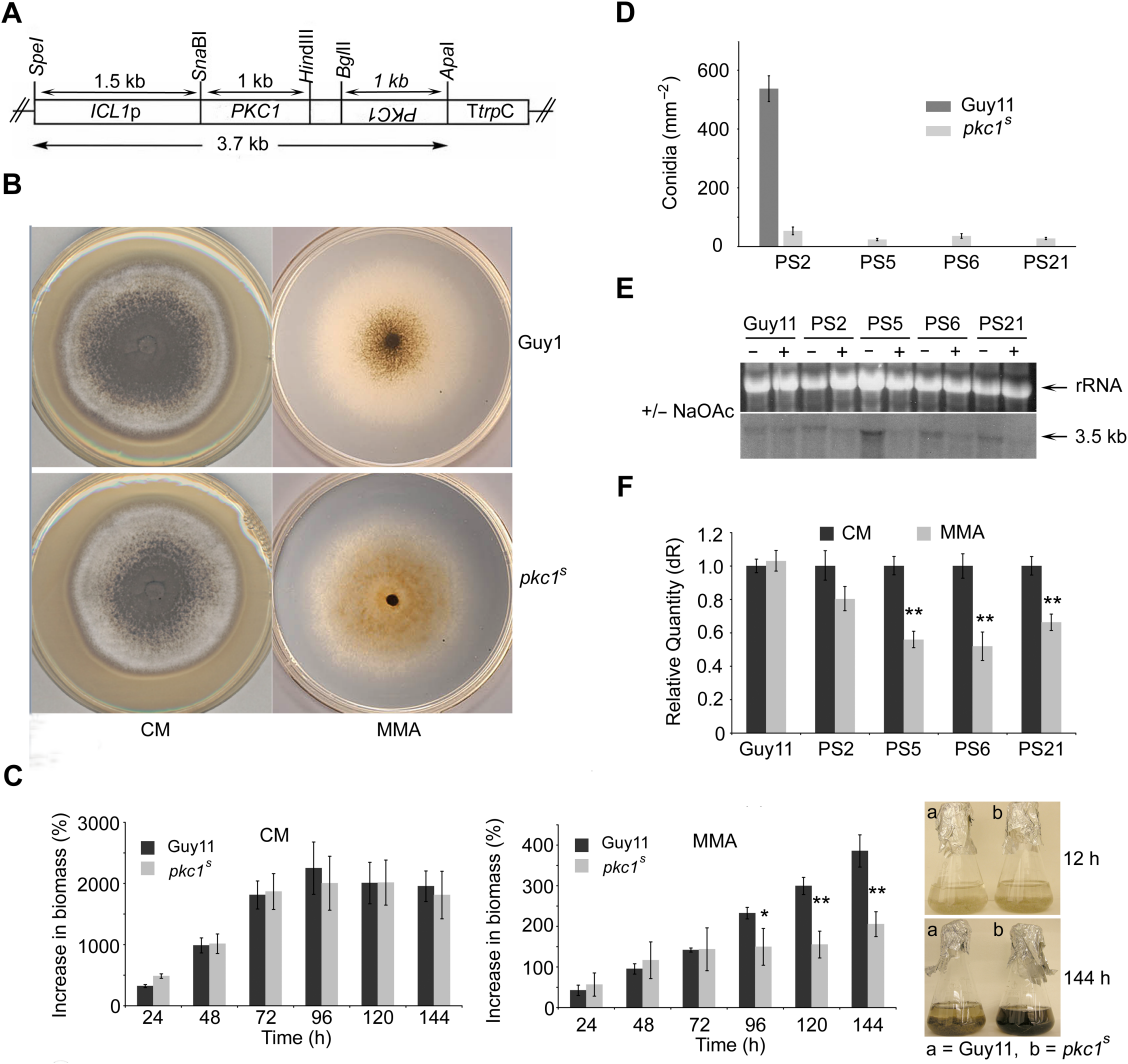
Functional characterisation of *PKC* *1* using an RNAi‐mediated gene silencing approach. A. The transcriptional unit for *PKC* *1* hairpin RNA expression under control of the acetate‐inducible *ICL* *1* promoter. B. *PKC* *1*‐gene silencing was induced in the presence of acetate (55 mM) as sole carbon source. Colonies revealed sparse hyphal growth and an orange/brown pigmentation. C. Bar charts to show biomass of wild‐type (Guy11) and *pkc1^s^* strains of *M* *. oryzae* following acetate induction of PKC gene silencing. Mycelium was harvested from CM (non‐inductive) or MM acetate (inductive) medium and dried. After 72 h induction, there was a significant reduction in biomass for the *pkc1^s^* strain compared with the wild type (*P < 0.05, **P < 0.01). Error bar represents standard deviation. Induction of silencing in the *pkc1^s^* strain caused excessive melanisation. D. Significantly fewer conidia were produced following the induction of *PKC* *1*‐gene silencing (Student's t‐test *P* < 0.005 for all strains). E. Northern blot analysis showing a reduction in *PKC* *1* mRNA transcript accumulation in silencing conditions (MMA). F. Comparative quantitative RT‐PCR analysis showing reduction in levels of *PKC* *1* in samples following induction of silencing (Student's t‐test **P < 0.01).

In order to test whether *PKC1* is essential for viability in *M. oryzae*, an attempt was made to replace the open reading frame of the gene with a selectable marker. In spite of repeated efforts utilising both Guy11 and a *Δku70* mutant lacking the non‐homologous DNA end‐joining pathway (Kershaw and Talbot, 2009), we were unable to recover any *Δpkc1* mutants. We therefore adopted an alternative strategy to downregulate expression of *PKC1* using RNA‐mediated gene silencing. RNA‐mediated gene silencing in fungi is initiated through the synthesis of dsRNA *in vivo* from a DNA template that is introduced and expressed in the fungus. For this study, we employed the silencing vector pSilent‐1 (Nakayashiki *et al*., 2005), which facilitates introduction of a hairpin dsRNA. The pSilent‐1 vector was first modified by introduction of the *ICL1* promoter from *M. oryzae* to replace the constitutive *Aspergillus nidulans trpC* promoter. *ICL1* encodes isocitrate lyase, an enzyme of the glyoxylate cycle and its expression is induced in the presence of acetate as sole carbon source and repressed when the fungus is grown in glucose‐rich medium (Wang *et al*., 2003). To induce *PKC1* RNAi‐mediated gene silencing, transformants carrying the transcriptional unit for *PKC1* hairpin RNA expression (Fig. 3A) were sub‐cultured onto minimal growth medium containing sodium acetate (55 mM) as sole carbon source (MMA). Of the transformants screened, 78% revealed a phenotype that was distinct from that observed in the wild‐type strain Guy11 under the same conditions, with colonies having a distinct brown pigmentation compared with the grey/green colouring of the wild type (see Fig. 3B). The colonies varied in the degree of pigmentation and their growth rate. We reasoned that this might be due to copy number or the site of random insertion of the gene silencing vector into *M. oryzae*. Four transformants, PS2, PS5, PS6 and PS21, hereafter referred to as *pkc1^s^* mutants, were selected for further analysis.

Induction of *PKC1* gene silencing resulted in less uniform radial growth, therefore hindering the accurate determination of colony size. Colonies revealed much sparser hyphal growth, so we compared the increase in biomass of *pkc1^s^* mutants with that of the wild type (Fig. 3C) over a period of 144 h. A similar increase in biomass for both strains was observed when cultured in liquid CM, but after 72 h in MMA, there was a significant reduction in biomass for the *pkc1^s^* strain compared with the wild‐type (*P* < 0.05 and P < 0.01 after 120 h). Upon induction of silencing, the mycelium and culture medium of the *pkc1^s^* strain became noticeably and progressively darker than Guy11 due to excess melanin production, as shown in Fig. 3C. The *pkc1^s^* mutants were also affected in their ability to produce conidia, with significantly fewer conidia produced compared with the wild type, Guy 11 (*P* < 0.005), as shown in Fig. 3D.

To determine whether there was a correlation between the observed changes in growth phenotype and the levels of *PKC1* expression, we examined *PKC1* mRNA accumulation. Northern blot analysis revealed lower levels of the *PKC1* transcript in the silenced transformants, as shown in Fig. 3E. Analysis by real‐time reverse transcription polymerase chain reaction (real‐time RT‐PCR) was employed to confirm the reduction in *PKC1* mRNA levels. A comparative quantitation experiment was carried out with total RNA extracted from hyphae grown in CM culture medium (repressing conditions). Expression levels of *PKC1* following induction of silencing were determined relative to the expression level in repressing conditions. After 48 h, all four mutants showed a significant reduction (*P* < 0.05) in *PKC1* mRNA levels (Fig. 3F). The reduction in *PKC1* transcript levels observed in transformants varied from 20 to 58% based on relative quantity compared with the control.

### Phenotypes of *pkc1^s^* mutants are due to RNA interference

**Figure 4:**
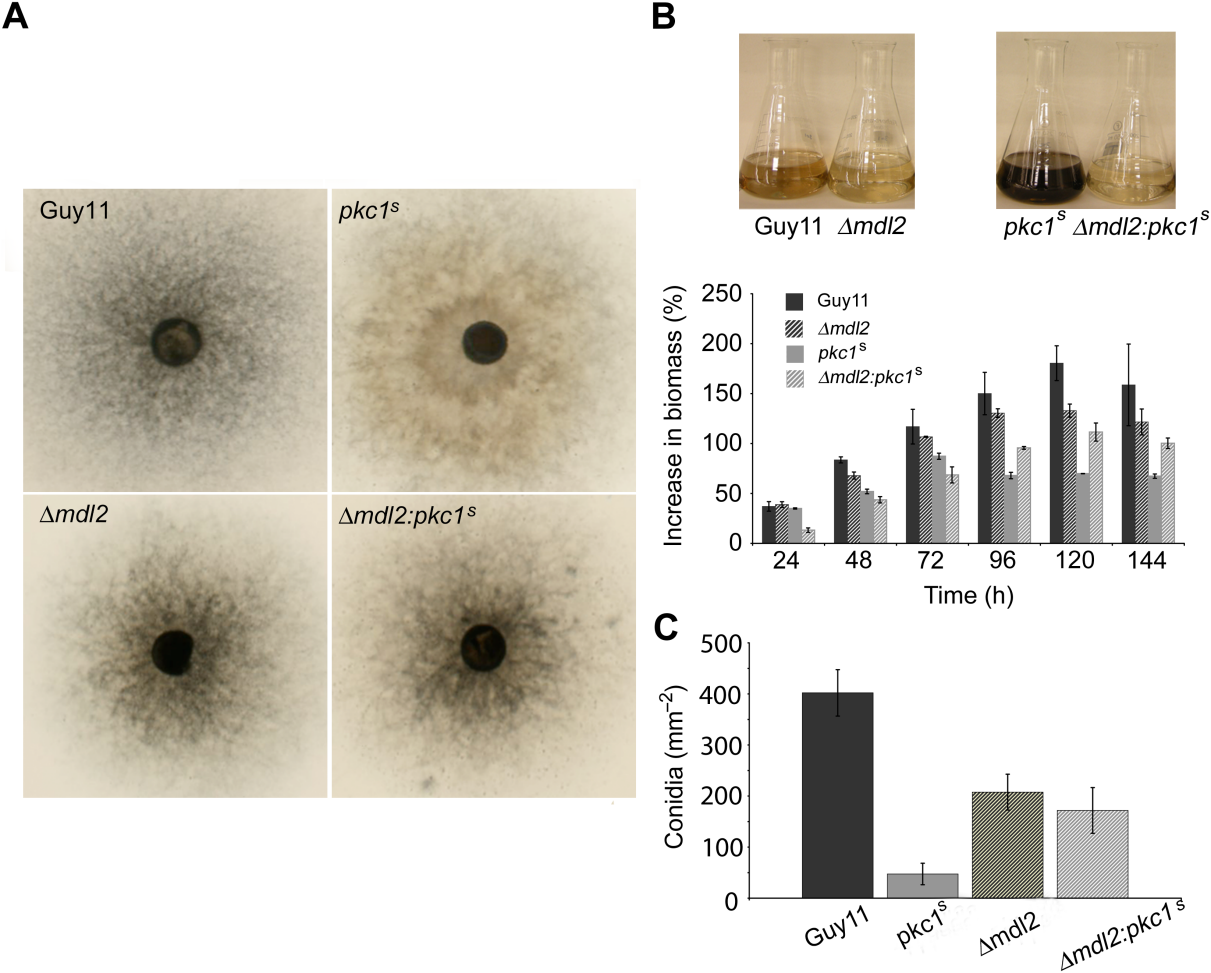
Targeted deletion of the Dicer‐like protein, Mdl2, in the *pkc1^s^* mutant strain complements the *pkc1* ^*s*^ mutant phenotype. A. Targeted deletion of *MDL* *2* in the wild‐type strain, Guy11, caused a reduction in the rate of growth compared with the parental strain. Targeted deletion of *MDL* *2* in a *pkc1^s^* strain resulted in dense hyphal growth and spore pigmentation compared with the *pkc1^s^* mutant. B. Bar charts to show biomass of Guy11, *Δmdl2*, *Δmdl2:pkc1^s^* and *pkc1^s^* strains of *M* *. oryzae* following acetate induction of *PKC* *1* gene silencing °C. Mycelium was harvested at the recorded time points and dried. After 72 h in MMA, the *Δmdl2:pkc1^s^* mutants produced significantly more biomass than the *pkc1^s^* strain (*P* < 0.05). Error bar represents standard deviation. Deletion of *MDL* *2* also prevented melanisation of cultures associated with *PKC* *1* silencing. C. The *Δmdl2:pkc1^s^* mutants produced significantly more conidia compared with the *pkc1^s^* strain (*P* < 0.05) and numbers were consistent with those produced following targeted deletion of *MDL* *2*. Error bar represents standard deviation.

In order to investigate whether the phenotypes ascribed to the *pkc1^s^* mutants were a direct result of a functional hairpin‐induced silencing process, we targeted the Dicer‐like genes *MDL1* and *MDL2*, which are components of the RNA‐mediated gene‐silencing pathway in *M. oryzae*. Consistent with previous research (Kadotani *et al*., 2004), *MDL1* was dispensable for RNA‐mediated gene silencing and *Δmdl1* transformants displayed the *pkc1^s^* phenotype following induction of *PKC1*‐gene silencing (not shown). However, targeted deletion of *MDL2* in the *pkc1^s^* strain resulted in denser hyphal growth, increased conidiation and wild‐type pigmentation. The phenotype of *Δmdl2:pkc1^s^* mutants was therefore comparable with that observed in the *Δmdl2* mutant or wild‐type strain, Guy11 (Fig. 4A). The colony size of *Δmdl2:pkc1^s^* mutants was, however, reduced compared with the wild type because targeted deletion of *MDL2* alone produces a similar reduction (Kadotani *et al*., 2004). Comparison of biomass revealed that targeted deletion of *MDL2* in either Guy11 or the *pkc1^s^* strain resulted in reduced growth compared with the wild type, but after 72 h, the *Δmdl2:pkc1^s^* mutants produced significantly more biomass (*P* < 0.05) than the *pkc1^s^* strain (Fig. 4B). Comparison of the culture medium after 144 h also revealed a striking difference following targeted deletion of *MDL2*, with no evidence of the excess melanin pigment formation observed in *Δmdl2:pkc1^s^* mutants. The *Δmdl2:pkc1^s^* mutants also produced significantly more conidia than the *pkc1^s^* strain (*P* < 0.05) and were consistent with conidiation of the *Δmdl2* mutant.

### A chemical genetic approach to targeted inhibition of *PKC* *1*

**Figure 5:**
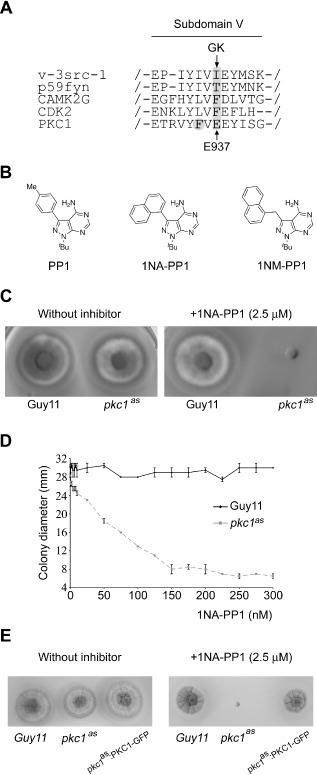
Selective kinase inhibition of protein kinase C in *M* *. oryzae*. A. Generation of an analogue‐sensitive mutant allele of *PKC* *1.* Partial sequence alignment of c‐Src and *PKC* *1* with other gate keeper‐substitution‐tolerant kinases. GK, gate keeper residue. B. Chemical structures of PP1 and C3‐derivatised analogues. C. A plug of mycelium from Guy11 and the *pkc1* ^as^ strain was inoculated onto medium containing 1NA‐PP1 (2.5 μM) and incubated for 12 days. D. The *pkc1^as^* mutant showed a dose‐dependent growth response to 1NA‐PP1. E. Reintroduction of a native copy of the *PKC* *1* allele restored resistance to PP1 inhibitor and viability.

As RNAi resulted in only a partial loss of *PKC1* expression, we sought to test whether complete elimination of protein kinase C activity would affect viability of *M. oryzae*. It has been established that a structurally conserved bulky residue (generally referred to as the gatekeeper residue), found in the ATP‐binding site of a kinase, is pivotal in determining sensitivity to the inhibitor PP1 (Bishop *et al*., 2000). Replacement of this residue with either alanine or glycine creates a pocket, which renders the kinase susceptible to chemically modified analogues of PP1. We identified the gatekeeper residue E937 from sequence alignment of *PKC1* with c‐Src (Fig. 5A) and replaced it with glycine (E937G). A second‐site mutation (Zhang *et al*., 2005), F935L, also, however, proved necessary for stabilisation of the kinase and successful recovery of the analogue‐sensitive *PKC1* allele (*pkc1* ^AS^), which retained the wild‐type phenotype in the absence of inhibitor. We synthesised two C3 PP1 moieties, 1NA‐PP1 and 1NM‐PP1 (see Supplementary Methods and Fig. 5B), to target the pocket created in the ATP‐binding site and tested the efficacy of these inhibitors by incorporating them into growth medium. 1NA‐PP1 (2.5 μM) abolished all growth from a plug of mycelium within 24 h with no observable effect on the wild‐type strain Guy11 (Fig. 5C). Both inhibitors caused a dose‐dependent decrease in colony size of the *pkc1* ^AS^ strain, while growth of the wild type remained relatively constant, with 1NA‐PP1 the more potent inhibitor (Fig. 5D). When a copy of the native *PKC1* gene was re‐introduced into the *pkc1* ^AS^ strain, susceptibility to the inhibitor was lost. The Pkc1:GFP plasmid was transformed into the *pkc1^AS^* mutant and transformants carrying single‐copy integrations were selected by Southern blot analysis (data not shown). The transformants were resistant to 1NA‐PP1 (2.5 μM) and viability was restored, as shown in Fig. 5E. We conclude that targeted inhibition of the kinase activity of protein kinase C is sufficient to prevent growth of the rice blast fungus.

### Global transcriptional profile analysis of a *pkc1^AS^* mutant following selective kinase inhibition

**Figure 6:**
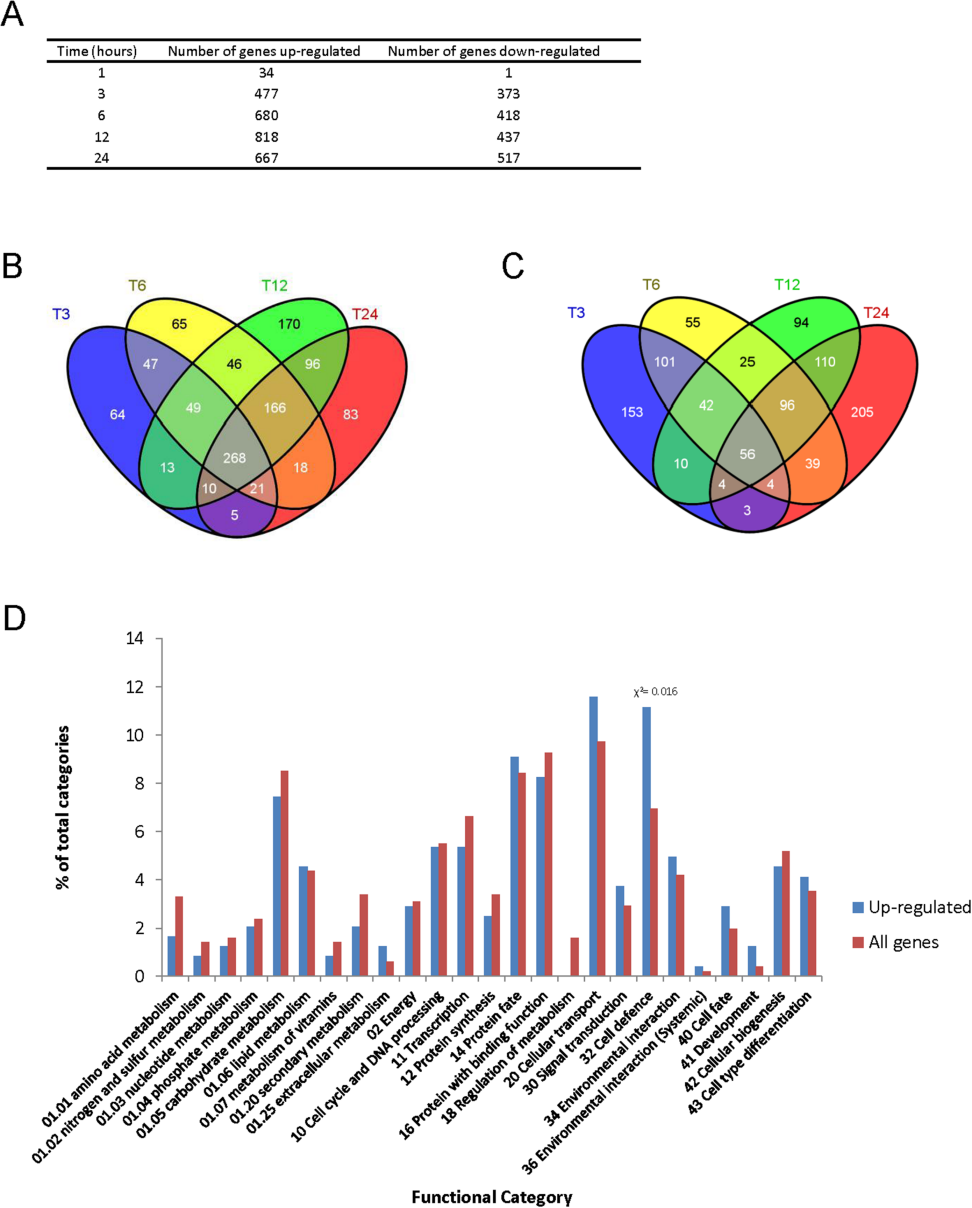
Global transcriptional profile analysis of *M* *. oryzae* by RNA‐Seq following selective protein kinase C inhibition. A. Table showing number of genes that show significant upregulation and downregulation (*P* < 0.01) following addition of 500 nM 1NA‐PP1 to the *pkc1* ^as^ mutant of *M* *. oryzae*. B. Venn diagram showing numbers of genes significantly upregulated (*P* < 0.01). C. Downregulated in the *pkc1* ^as^ mutant after exposure to 1NA‐PP1 for 3, 6, 12 or 24 h. D. Bar chart showing the number of functional categories represented in the 268 genes significantly upregulated upon 1NA‐PP1 exposure at all times. A χ^2^ test (using Yates correction) was used to identify functional categories that show significant over‐representation in upregulated gene set when compared with total genome. Genes allocated to functional categories are based on the FunCat schema (Ruepp *et al*., 2004).

In order to define the global effect on gene expression of selectively inhibiting PKC activity in *M. oryzae*, RNA‐seq analysis was performed. The *pkc1^AS^* mutant was grown for 24 h in the presence or absence of 1NA‐PP1 and total RNA extracted at 1, 3, 6, 12 and 24 h. RNA‐seq analysis was then performed using two biological replicates per time point (Table S1). The number of genes showing significant (P‐value < 0.01) differences in gene expression was recorded at each time point (Fig. 6A). Very few genes showed differential regulation 1 h after the addition of 1NA‐PP1 (34 were upregulated and only 1 downregulated), but Euclidian distance analysis between expression data from each time point revealed significant divergence in gene expression patterns after 3 h exposure with 477 genes upregulated and 373 downregulated (See Fig 6 and Fig. S4). Analysis of gene expression across the 24 h period of exposure revealed 268 genes to be differentially upregulated at all time points between 3 h and 24 h, from a total of 1122 genes showing significant upregulation in at least one time point (Fig. 6B). A much smaller number of genes showed differential downregulation at all time points, 56 from of a total of 1746 genes that show significant downregulation in at least one time point (Fig. 6C). Differentially expressed genes were classified using the FunCat schema (Ruepp *et al*., 2004) into putative functional categories (Fig. 6D). A single functional category *Cell Defence* was over‐represented among upregulated genes. This group includes genes involved in stress response and cell death, consistent with the loss of viability caused by inhibition of PKC.

**Figure 7:**
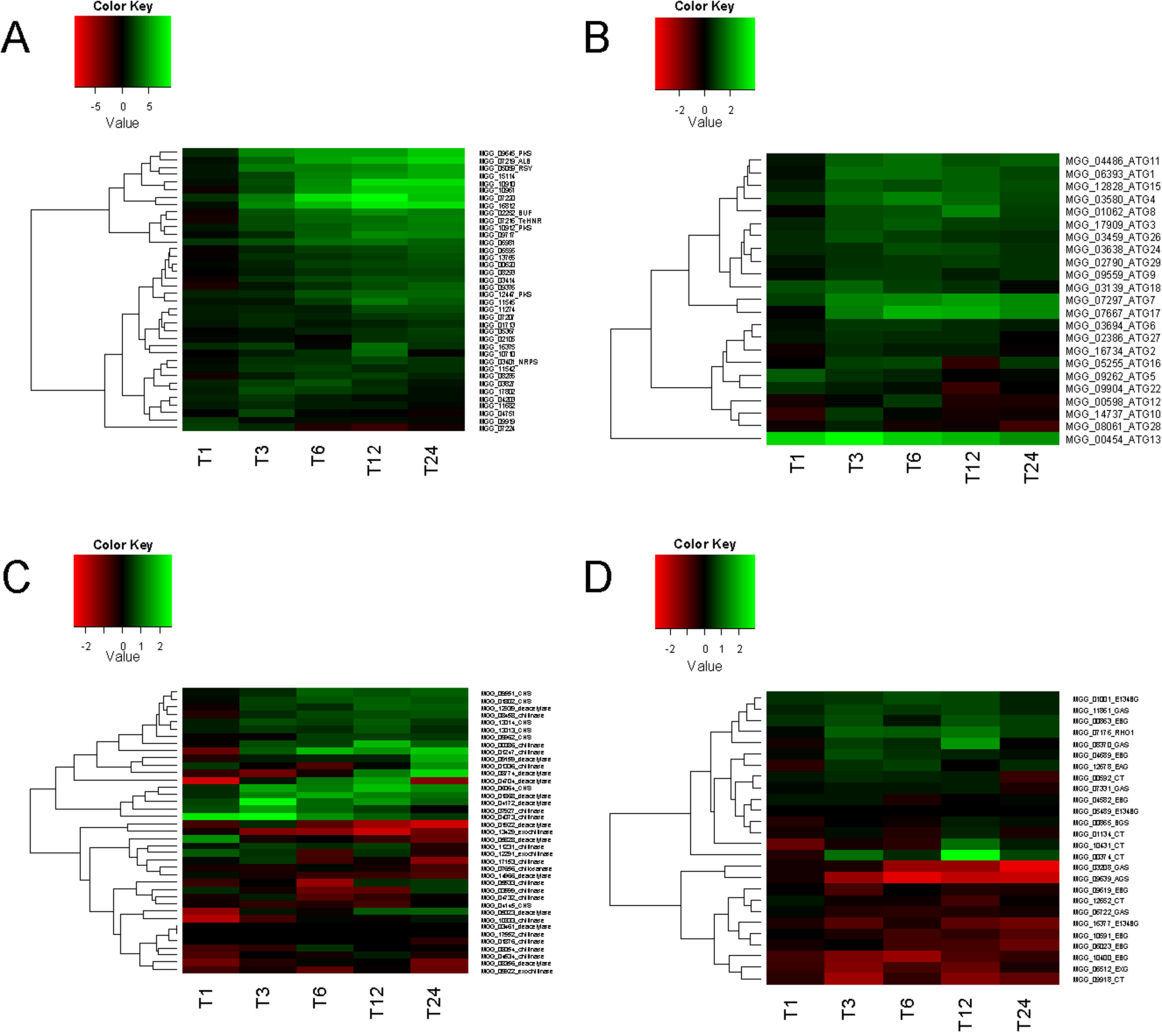
Heatmaps showing log_2_ expression ratio of functionally related gene sets following selective protein kinase C inhibition of the *pkc1* ^as^ mutant of *M* *. oryzae* with 1NA‐PP1. A. Genes involved in secondary metabolism significantly upregulated (P < 0.01) in at least one time point. Components of the melanin biosynthetic pathway labelled (*ALB* *1*, *BUF* *1*, *RSY* *1*, *THNR* are labelled) as well as polyketide synthases (PKS) and non‐ribosomal peptide synthases (NRPS). B. Genes encoding autophagy‐associated proteins. C. Genes encoding enzymes involved in chitin biosynthesis and re‐modelling (CHS, chitin synthase). D. Genes encoding enzymes involved in glucan biosynthesis and re‐modelling (BGS, 1,3‐beta‐d‐glucan synthase catalytic subunit; RHO1, 1,3‐beta‐d‐glucan synthase regulatory subunit; CT, chitin transglycosylase; EXG, exo‐1,3‐beta‐glucanase; EBG, endo‐beta‐1,3‐glucanase; E134BG, endo‐1,3(4)‐beta‐glucanase; GAS, beta‐1,3‐glucanosyltransferase; AGS, alpha‐1,3‐glucan synthase; EAG, endo‐1,3‐alpha‐glucosidase).

To investigate the cellular processes regulated by PKC, a systematic analysis of differentially expressed genes was carried out and heatmaps were constructed based on the log_2_ expression ratio between 1NA‐PP1‐treated and untreated samples. Genes were then functionally assigned and the data were analysed based on putative cellular processes being affected (Fig. 7). A heatmap of genes predicted to be involved in secondary metabolism showed significant upregulation of large sets of genes in the presence of 1NA‐PP1‐treated mycelium in at least one time point. The melanin biosynthetic pathway (Howard and Valent, 1996), for example, shows upregulation from 3 h (Fig. 7A), consistent with observed melanisation of mycelium following *PKC1* silencing (Fig. 3C). A putative polyketide synthase, three FAD‐binding domain‐containing protein‐encoding genes (MGG_15114, MGG_16812, MGG_10961), a homologue of an aflatoxin biosynthesis ketoreductase (MGG_10910) and an iron transport multicopper oxidase (MGG_07220) clustered with the *ALB1* and *RSY1* genes based on expression profile (Fig. 7A). The *BUF1* and *THNR1* genes clustered with a second polyketide synthase, a potential gluco‐oligosaccharide oxidase/chitin‐binding protein (MGG_09717) and a carnitine acetyl transferase (MGG_06981), suggesting that secreted and cell wall‐associated secondary metabolites, synthesised by these routes, are expressed in response to loss of PKC activity. Similarly, genes associated with non‐selective autophagy (Kershaw and Talbot, 2009) collectively showed upregulation after 3 h exposure to 1NA‐PP1 (Fig. 7B) particularly *ATG7*, *ATG13* and *ATG17*, consistent with severe cellular stress being imposed by PKC inhibition, leading to triggering of autophagic recycling as a cell survival response.

Expression of genes involved in fungal cell wall biogenesis and re‐modeling showed significant differential regulation in response to PKC inhibition, consistent with the role of PKC in cell integrity (Paravicini *et al*., 1996). A number of genes involved in chitin synthesis, for example, were shown to be upregulated upon PKC inhibition, including six chitin synthases, three endochitinases and two chitin deacetylases (Fig. 7C). Interestingly, a large set of cell wall‐associated glucan biosynthetic genes were upregulated as a response to loss of PKC activity, while a second clade showed significant downregulation, consistent with them being associated with *PKC1*‐regulated normal growth and development. Among the 1NA‐PP1‐repressed gene functions were a family of endo‐beta‐1,3‐glucanases, such as MGG_10400.

To investigate the relationship of the differentially expressed genes in *M. oryzae* following 1NA‐PP1 treatment to those associated with the cell integrity pathway, we classified all known homologues of genes previously demonstrated in *S. cerevisiae* to be members of this pathway (Garcia *et al*., 2004). In yeast, a total of 178 genes have been identified to be upregulated following induction of cell wall damage with Congo Red and Zymolyase. This upregulation is dependent on the MAP kinase, Slt2 and the transcription factor Rlm1 in nearly all cases, with these proteins acting downstream of Pkc1 (Garcia *et al*., 2004). We identified 93 homologues of these genes in *Magnaporthe oryzae* based on best‐bidirectional BLASTP hits (with an e‐value cut‐off of 10^−5^). Expression ratios in 1NA‐PP1 treated mycelium of the *pkc1^AS^* mutant compared with uninhibited mycelium are shown in Fig. S5. A group of 26 genes showed upregulation during 1NA‐PP1 inhibition of the *pkc1^AS^* mutant. Five of these are involved in cell wall organisation and biogenesis, three of which are involved in chitin biosynthesis. A further six are involved in metabolism/energy, though from different metabolic pathways. This clade also included three drug transporters (PDR5 homologues, described in Golin *et al*., 2007). The homologue of *SLT2* was found in this clade as well as a homologue of *MSG5*, a dual‐specificity protein phosphatase‐encoding gene involved in the cell integrity pathway and regulated by Slt2 in *S. cerevisiae* (Flández *et al*., 2004). A clade of 18 genes showed downregulation during Pkc1 inhibition. Two of the downregulated genes are involved in energy production: cytochrome c oxidase and a citrate synthase. There were also three genes involved in cell wall metabolism: a glucanosyl transferase, an exo‐β‐1,3 glucanase and a chitin transglycosylase.

Annotation of the 268 genes showing significant upregulation at all time points between 3 and 24 h following 1NA‐PP1 treatment identified other related gene functions (Table S2), such as 22 putative transcription factors, of which 5 had previously been identified as being upregulated during conidiation and 2 upregulated during appressorium development and germination (Park *et al*., 2013). In addition, an N‐6 adenine‐specific DNA methyltransferase identified as being highly expressed *in planta* was observed (Kim *et al*., 2010), as well as a large set of genes involved in cell stress responses. A total of 19 proteolysis‐associated genes, including 7 genes in the ubiquitin modification pathway, as well as 8 genes encoding potential F‐box proteins. There were also 15 genes involved in signal transduction, including 2 protein kinases, one of which was *MPS1*, encoding the MAP kinase involved in the cell integrity pathway that is a functional homologue of *SLT2* in yeast (Xu *et al*., 1998) and a pH response regulator homologous to *palA* from *Aspergillus nidulans* (Negrete‐Urtasun *et al*., 1997), three G‐protein coupled receptors, two with CFEM domains (Kulkarni *et al*., 2005), one protein phosphatase and two phospholipase genes, as well as 10 transporters, 6 of which were from the major facilitator superfamily. The extensive role of PKC in calcium signalling could also be observed based on broad classification of two groups of differentially regulated genes, either upregulated or downregulated by PKC inhibition (Fig. S6).

When considered together, the upregulated gene set pointed to extensive cell wall re‐modelling, secondary metabolism and cellular stress responses, such as autophagy and regulated proteolysis, resulting from loss of PKC, with many, but not all functions associated with stimulation of the cell integrity pathway. By contrast, repressed gene families included those associated with cell wall re‐modelling functions, that may be growth associated, and a large set of calcium signalling gene functions.

In order to determine if the major cause of the loss of viability of the *pkc1^AS^* mutant in the presence of 1NA‐PP1 was inhibition of the cell integrity pathway, we carried out an experiment to see if we could maintain viability by osmotic stabilisation. Mutants in the cell integrity pathway in yeast are often osmotically remedial with their growth restored under hyper‐osmotic conditions (Flández *et al*., 2004). To do this, we incubated the *pkc1^AS^* mutant on growth medium supplemented with either 0.6 M of NaCl or 1.0 M of sorbitol, and observed if this led to restoration of viability in the presence of 1NA‐PP1. We found that no growth recovery could be elicited under these conditions (Fig. S7). We conclude that protein kinase C in *M. oryzae* is necessary for cellular viability based on wider regulatory functions than the Mps1‐dependent cell integrity pathway.

## Discussion

Fungal pathogens are difficult to control in an effective and durable manner. Resistance to fungicides, most notably the widely deployed triazoles (Verweij *et al*., 2007) and the more recently developed strobilurins (Fraaije *et al*., 2002), for example, means that new anti‐fungal agents are urgently required both for medical and agricultural applications. Only by deploying both more effective fungicides and durably disease‐resistant crop varieties will it prove possible to limit plant disease and make a significant contribution towards global food security.

The motivation for this study was to identify signalling pathways essential for viability of an economically devastating plant pathogen, the rice blast fungus *Magnaporthe oryzae*. Specifically, we set out to determine whether protein kinase C in *M. oryzae* is required for growth and development. We identified a single protein kinase C‐encoding gene in the rice blast fungus, which appears typical of filamentous fungi. Protein kinase C isozymes in mammalian systems have distinct physiological roles and differ in activator and substrate preferences accordingly. The isoforms are classified as classical (cPKC), novel (nPKC) or atypical (aPKC) according to their structure, which varies mainly in the regulatory domain. The sole PKC in *M. oryzae* is defined by an extended regulatory domain (Jacoby *et al*., 1997), which may well compensate for the lack of additional isoforms and is consistent with the kinase potentially serving multiple functions and contributing to diverse signalling networks.

Evidence from our initial pharmacological studies suggested that PKC activity is required in the early stages of spore germination and appressorium development and no effect was observed when PKC‐inhibiting drugs were added 6 h post‐incubation, for example. This is interesting because by this stage *M. oryzae* germlings are committed to appressorium differentiation, a process for which DNA replication and entry into mitosis are both pre‐requisites (Veneault‐Fourrey *et al*., 2006; Saunders *et al*., 2010). Based on our initial chemical analysis, we speculated that PKC activity may regulate processes such as cell cycle regulation and morphogenesis, in addition to its well documented role in the cell wall integrity pathway in fungi (Heinisch *et al*., 1999).

To test the function of *PKC1* in *M. oryzae*, we initially attempted targeted gene replacement but this did not result in any null mutants, in spite of numerous attempts including utilisation of a *Δku70* strain with enhanced frequency of homologous recombination (Kershaw and Talbot, 2009). We therefore decided to attempt gene silencing by means of RNA interference (RNAi), which has been reported in *M. oryzae* (Nakayashiki *et al*., 2005) and results in downregulation of gene expression, rather than complete loss of gene function. RNAi provides a useful tool for functional analysis of putatively essential genes. The utility of RNAi in *M. oryzae* (and most other fungal pathogens) is, however, presently limited by the paucity of inducible promoters, with well‐evidenced levels of repression. In our study, we used the glucose‐repressible, acetate‐inducible promoter from the isocitrate lyase‐encoding gene *ICL*1 (Wang *et al*., 2003). This promoter is of limited utility because acetate is not a good carbon source for the fungus and results in more sparse growth even by wild‐type strains. We observed that downregulation of *PKC1* expression was accompanied by significant impairment in fungal growth and reduction in biomass and sporulation, compared with the isogenic wild‐type strain Guy11. We did not, however, observe specific effects related to germination and appressorium formation, in contrast to inhibitor studies, but the conidia used in these assays were obtained from conditions in which the RNAi construct was not expressed because insufficient conidia could be obtained from medium containing acetate. Although acetate was added to conidia in these experiments to induce silencing, it is possible that there was sufficient *PKC1* transcript present to facilitate both germination and appressorium differentiation. Gene silencing of *PKC1* nevertheless provided further evidence of the importance of the kinase to cellular viability, as colonies grow poorly and there was an intense autolytic phenotype, visible particularly in liquid culture where melanin leached into the medium (Fig. 3C). This is consistent with PKC serving an important function in cell wall integrity, which appeared compromised under conditions of silencing. Verification of gene silencing was achieved by deleting the Dicer 2‐encoding gene, *MDL2*, which remediated all phenotypes associated with *PKC1* silencing. This provides arguably the most robust control experiment for any phenotype associated with a gene silencing experiment in *M. oryzae*.

In view of the problems associated with gene silencing experiments and lack of a viable gene deletion mutant, we sought an independent method to demonstrate the role of *PKC1*. We therefore generated an analogue‐sensitive allele of the protein kinase, which would be susceptible to the kinase inhibitor PP1. This can be achieved by mutation of the bulky gatekeeper residue of the ATP‐binding pocket of a kinase, with alanine or glycine, providing enhanced binding of the inhibitor in a selective manner (Bishop *et al*., 2000). We found that a second site mutation was necessary to stabilise the kinase, but the synthetic allele generated and introduced, by homologous recombination, into the fungus proved highly sensitive to PP1. In this way, we were able to provide strong evidence for the role of *PKC1* in cellular viability as complete loss of growth occurred when the inhibitor was added.

Transcriptional profile analysis demonstrated the global nature of PKC‐mediated gene regulation in *M. oryzae* with a total of 2868 genes, or 26% of the predicted genome (Dean *et al*., 2005), showing differential expression upon PKC inhibition. PKC inhibition led to significant effects on cellular processes, such as calcium signalling, autophagy, signal transduction and transcriptional regulation with 22 transcription factors among the differentially expressed gene sets. The role of PKC is therefore likely to be wider than the predicted known roles in regulation of the cell integrity pathway (Heinisch *et al*., 1999), which may explain why it is necessary for cellular viability. The other components of the cell integrity pathway, for example, such as the *MPS1* MAP kinase and the *BCK1/MCK1* MAPKKK, are clearly not essential because null mutants can be readily recovered (Xu *et al*., 1998; Jeon *et al*., 2008).

To test whether the regulation of the cell integrity pathway by *M. oryzae* by PKC was the main reason for its role in cellular viability, we carried out two specific experiments. First, we investigated the degree to which the transcriptional regulation based on loss of PKC function in an analogue‐sensitive mutant mirrored the well‐known components of the cell integrity pathway. We observed a relatively small set of only 26 known components of the cell integrity pathway showing upregulation after PKC1 inhibition, including five genes involved in cell wall organisation and biogenesis, three of which are involved in chitin biosynthesis. Furthermore, 18 of the downregulated genes were also reported components of the cell integrity pathway by comparison with budding yeast (Garcia *et al*., 2004). However, the global transcriptional response in yeast involves 178 genes that are specifically upregulated (Garcia *et al*., 2004), so the degree of overlap was not as large as might have been expected. Furthermore, cell integrity pathway mutants in yeast, and other fungi, are often known to be osmotically remedial – that is, their mutant phenotypes can be complemented by the presence of osmotically stabilised growth medium (Heinisch *et al*., 1999; Flández *et al*., 2004). We observed that the loss of cellular viability of the *pkc1^AS^* mutant could not be restored under hyperosmotic conditions in the presence of 1NA‐PP1, consistent with PKC in *M. oryzae* having a much wider set of potential regulatory targets. These clearly include not only calcium signalling pathways, consistent with a recent study in *A. nidulans* (Colabardini *et al*., 2014), secondary metabolic pathways, autophagy‐associated genes, but also a more complex transcriptional regulation of cell wall biogenesis. Complex regulation of glucan biosynthetic genes, for example, occurs with examples that are both induced and repressed by inhibition of PKC in *M. oryzae*. Further analysis of this response may identify which of these glucan biosynthetic pathways are associated with normal hyphal growth, branching and sporulation, and which are specifically necessary to ensure cell viability under conditions of severe stress. Clearly, PKC plays a key role in the balance of these responses and the homeostatic control of cell wall biogenesis, which is wider than the known cell integrity control pathway predicted from *S. cerevisiae*.

The loss of viability in the *pkc1^AS^* mutant in the presence of 1NA‐PP1 and the extent of the transcriptional response of *M. oryzae* to PKC inhibition clearly show that *PKC1* must therefore act upstream of many additional components regulating pathways other than the cell integrity response in the fungus, providing evidence of its efficacy as a potential anti‐fungal drug target. The analogue‐sensitive mutant and its rapid response to 1NA‐PP1 clearly demonstrates that PKC inhibition is a rapid and effective way of halting growth and viability of the rice blast fungus, which may have important consequence for disease control strategies.

## Experimental procedures

### Growth and maintenance of *M* *. oryzae*

Fungal isolates used in the study are stored at the University of Exeter. Storage of the fungus, preparation of growth medium and DNA‐mediated transformation were all carried out as reported previously (Talbot *et al*., 1993). Restriction enzyme digestion, gel electrophoresis, and DNA and RNA gel blot hybridisations were performed using standard procedures (Sambrook *et al*., 1989).

### Pharmacological inhibition of PKC

All PKC inhibitors used in this study were purchased from Sigma. A stock solution of Ro‐31–8220 (1 mg ml^−1^, 1.8 mM) was prepared in ddH_2_O and stored in aluminium foil wrapping at 4°C. A stock solution of chelerythrine chloride (1 mg ml^−1^, 2.6 mM) was made in dimethyl sulfoxide (DMSO) (100%) and stored at −20°C.

PP1 and the analogues, 1NA‐PP1 and 1NM‐PP1, were synthesised as previously described (Hanefeld *et al*., 1996, see Supplementary Methods). Stock solutions (10 mM) were prepared in DMSO (100%) and stored at −20°C. Prior to use, the inhibitors were diluted in ddH_2_O to the required concentration.

### Assays for sporulation, conidial germination and appressorium formation

Conidial germination and the development of appressoria were monitored over time on a borosilicate glass coverslip, using a method adapted from Hamer *et al*. (1988). Strains were grown on CM agar for 12 days at 24°C. A conidial suspension of 5 × 10^4^ conidia ml^−1^ was prepared in ddH_2_O and 50 μl was pipetted onto the surface of the coverslip. Following incubation in a moist chamber at 24°C, 300 conidia were counted and the percentage that had undergone the stated developmental event was recorded.

### Construction of the *PKC* *1*–*GFP* gene fusion

For construction of the *PKC1:sGFP* gene fusion, a 5.9 kb of genomic fragment containing the *PKC1* coding region (excluding the translation stop codon) and 2 kb of upstream promoter sequence was amplified with primers PKC.GFP.F50, 5′‐TAGAATTCCCAGGCTAGACTAGACTATG‐3′ and PKC.GFP.R30, 5′‐TACCATGG **T**ATCAAAGTCTGCCGTGTA‐3′, modified to introduce an *Eco*RI and *Nco*I restriction sites (underlined) at the 5′ and 3′ ends of the genomic fragment respectively. An additional base (shown in bold) was incorporated into the 3′ primer to ensure that the gene fusion was in‐frame with the translation start codon of the sGFP allele. The fragment was inserted into *Eco*RI/*Nco*I digested pMJK142.2 plasmid, which contains a fungal codon‐optimised synthetic allele of the *GFP* reporter gene. The *M. oryzae ILV1* gene conferring sulfonylurea resistance was amplified from plasmid pCB1532 using primers designed to introduce *Eco*RI restriction sites at either end to allow insertion into the *Eco*RI restriction site within the PKC–GFP construct. Following DNA sequence analysis to confirm that the gene fusion was in‐frame, the construct was introduced into Guy11, the wild‐type strain of *M. oryzae*. Transformants were selected using chloromuron ethyl (50 μg ml^−1^) and confirmed by DNA gel blot hybridisation.

### Construction of silencing vector

To construct the *ICL1p*:*pkc1^s^* conditional gene silencing vector a 1 kb fragment of the *PKC1*‐encoding gene was amplified using the primer pairs PKC_SnaBI 5′‐GCTACGTAGAAGCCCCTTACCGGTCAATTATC‐3′ (*Sna*BI site underlined) and PKC_HindIII 5′‐GCAAGCTTTTGACACTCGCTGCATTTTCTGCA‐3′ (*Hin*dIII site underlined) for insertion in the sense orientation and PKC_ApaI (*Apa*I site underlined) 5′‐TAGGGCCCGAAGCCCCTTACCGGTCAATTATC‐3′ and PKC_BglII 5′‐GCAGATCTTTGACACTCGCTGCATTTTCTGCA‐3′ (*Bgl*II site underlined) for insertion in the antisense orientation. The respective fragments were inserted into the *Sna*BI/*Hin*dIII and *Bgl*II/*Apa*I sites in the multiple cloning site of pSilent‐1 (Nakayashiki *et al*., 2005) sequentially. The *A. nidulans trpC* promoter was excised by restriction digest using enzymes *Spe*I and *Sna*BI and replaced with the *M. oryzae ICL1* promoter which was amplified and modified by the addition of *Spe*I and *Sna*BI restriction sites using primers ICLI_SpeI.5′‐GCACTAGTGAATTCGTCCAGTAATCAAAGGCA‐3′ (*Spe*I site underlined) and ICLI_SnaBI 5′‐GCTACGTACTCGGGAATATGGTTCTTACGACA‐3′ (*Sna*BI site underlined). The construct was introduced into Guy11, the wild‐type strain of *M. oryzae*. The vector carries a hygromycin B resistance gene cassette for selection.

### Phenotypic analysis of *pkc1^s^* mutants

Vegetative growth was assessed from plate cultures grown on minimal medium containing acetate, 55 mM, as sole carbon source (MMA). Colony diameter was measured and recorded at 48 h intervals over a period of 12 days. To measure biomass, an equivalent amount of fungal mycelium from the Guy11 and mutant strain was inoculated into 500 ml liquid CM (Talbot *et al*., 1993) and incubated at 24°C, 125 rpm for 48 h. Mycelium was then filtered through Miracloth (Calbiochem, San Diego, USA), blotted dry and weighed. The mycelium from each strain was divided into 13 equal parts and transferred to fresh flasks, 6 flasks containing CM (150 ml) and 6 flasks with MMA (150 ml). Mycelium was incubated at 24°C, with shaking at 125 rpm and harvested after 24, 48, 72, 96, 120 or 144 h. The remaining portion and subsequent harvestings were wrapped in weighed aluminium foil and placed in a drying oven at 70°C until no further decrease in weight could be determined.

### Northern blot analysis

Total RNA was extracted from transformants grown in liquid CM for 48 h and for a further 24 h in either CM or MMA. Following fractionation by gel electrophoresis, the RNA was transferred to Hybond‐N (Amersham) and subsequently probed with a 1 kb fragment of *PKC1*, the sequence of which is located downstream (3′) of the fragment used in the transcriptional unit for *PKC1* hairpin RNA expression.

### Quantitative RT‐PCR

Following treatment with DNase1 (Invitrogen) total RNAs were reverse transcribed into first‐strand cDNA using the AffinityScript QPCR cDNA Synthesis Kit (Stratagene), according to the manufacturer's instructions. Real‐time quantitative PCR was performed using a MxPro‐Mx3005P system (Stratagene) using SYBR green I. Reaction mixtures were prepared as follows: 12.5 μl of brilliant SYBR Green I master mix, 12.5 ng cDNA, primers and nuclease‐free water to a final volume of 25 μl. Primers Pkc‐rtF50.1,5′‐ AAGCTCTATGAGTGACCGCACGTT‐3′ and Pkc‐rtR30.1, 5′‐ AGGTGATCCGCTGAGGTGAAGTTT were designed to amplify a fragment of 178 nt and added to the reaction mixture to a final concentration of 150 nM. The thermal profile included an activation step, of 95°C for 10 min, followed by 40 cycles of amplification. Cycling conditions consisted of denaturation at 95°C for 30 s, annealing at 56°C for 60 s and extension at 72°C for 30 s. Following amplification, a dissociation curve was generated to allow differentiation between specific and non‐specific amplicons (data not shown). The *TUB2* β‐tubulin‐encoding gene from *M. oryzae* was used in the assay as a normaliser. Reactions were prepared with three technical replicates per sample and experiments were routinely performed three times from different biological materials. Standard curves were generated to establish actual amplification efficiency values for both the gene of interest and the normaliser (data not shown). Fluorescence was monitored by the Mx3005P Real‐Time PCR system (Stratagene) software which employs an efficiency‐corrected enhancement of the 2*^−^* ^ΔΔ^ *^Ct^* quantification method (Livak and Schmittgen, 2001) for comparative comparison.

### Targeted gene replacement

All targeted gene deletions were carried out using a PCR‐based, split‐marker deletion method (Yu *et al*., 2004). Gene‐specific primers (see Table S3) were designed to allow amplification of ∼ 1 kb fragments from the 5′ and 3′ flanks of the open reading frame of the target gene. Primers for the inner flanks were designed to include an extension that was complementary to the ends of a fragment of a selectable marker to allow fusion to the marker in a second round of PCR. The hygromycin resistance (HYG) cassette was employed to attempt to generate a*PKC1* deletion strain (see Supplementary Methods and Fig. S3), *MDL1* was replaced by the phosphinothricin acetyltransferase cassette (BAR) and *MDL2* by the sulfonylurea resistance gene (Sweigard *et al*., 1997). Selection medium contained hygromycin B, 200 μg ml^−1^ (Calbiochem), glufosinate, 30 μg ml^−1^ or chlorimuron ethyl, 50 μg ml^−1^. Putative transformants were confirmed by DNA gel blot hybridisation with DNA extracted from fungal mycelium as described previously (Talbot *et al*., 1996).

### Construction of the *pkc1^AS^* allele

Polymerase chain reaction‐based mutagenesis was used to generate F935L and E937G mutations in the *PKC1* gene. Replacement of Phe‐935 with Leu required a T–C transversion at position 2965 and an A–C transversion at position 2972 substituted a glycine residue for glutamate‐937. The base changes were incorporated into complementary forward and reverse primers, sogg‐R30.45′‐ AAT GTA TTC TCC CAC GAG GTA TAC **C**CG**G**GT CTC‐3′ and sogg‐F50.45′‐GAG AC**C**CG**G** GTA TAC CTC GTG GGA GAA TAC ATT‐3′ (base changes underlined). The primers were designed to include two additional silent point mutations (bases shown in bold) to introduce a unique *Xma*I restriction site so facilitating analysis of putative transformants. In the first round of PCR, primer pairs Pkc‐EcoRI‐F50.1 5′‐TAGAATTCATGGATGACAGGATACAAGACA‐3′ (introducing an *Eco*RI restriction site, underlined) and sogg‐R30.4 and sogg‐F50.4 and Pkc‐XbaI‐R30.1 5′‐GCTCTAGAATGGATACTCTCCAGCTCAAAC‐3′ (introducing an *Xba*I restriction site, underlined) were used to amplify the open reading frame (plus the 3′ UTR) in two parts, 3 and 1.4 kb respectively. These fragments were then used as the template in a second round of PCR with primers Pkc‐EcoRI‐F50.1 and Pkc‐XbaI‐R30.1. The hygromycin resistance gene cassette was amplified from pCB1004 using primers Hyg‐Xba1F50.1 5′‐GCTCTAGAGGAGGTCAACACATCAATG‐3′ and Hyg‐Xma1R30.1 5′‐TACCCGGGCTCTATTCCTTTGCCCTCG‐3′ modified to introduce *Xba*I and XmaI restriction sites, shown underlined. To enable insertion of the selectable marker by homologous recombination, a 1 kb fragment downstream of *PKC1* was also amplified, using primers Pkc‐UTRF50.1 5′‐ TACCCGGGCATCCAGACGTGATTACATTG‐3″ (introducing an *Xma*I restriction site, shown underlined) and Pkc‐UTRR30.1 5′‐ TAGAATTCACGACCTGACGGGCTACT‐3′ (introducing an *Eco*RI restriction site, shown underlined). The three gene fragments were independently ligated into an intermediate vector, p‐GEM‐T (Promega), generating plasmids pTP144, pTP144.2 and pTP144.4 respectively. For alignment of the hygromycin‐resistant allele and the 1 kb fragment downstream of *PKC1*, plasmids pTP144.2 and pTP144.4 were digested with *Xma*I and *Not*I (located in the MCS of pGEM‐T), and the genomic fragment downstream of *PKC1* was inserted into pTP144.2 to generate plasmid pTP144.6. The hygromycin‐resistant allele and the 1 kb extension were excised as a 2.4 kb of *Xba*I–*Not*I fragment which was inserted into *Xba*I and *Not*I, digested pTP144 to form *PKC1^F935L,E937G^*. The *PKC1^F935L,E937G^*‐ encoding gene plasmid was then digested with *Eco*RI to give a 6.8 kb of fragment which was transformed into the wild‐type Guy11 strain.

### Transcriptional profile analysis

Mycelium of the *M. oryzae pkc1^AS^* mutant was prepared in CM shaking cultures at 24°C, 125 rpm for 48 h. Mycelium was then filtered through Miracloth, blotted dry and weighed. The mycelium was divided and treated with 1NA‐PP1, 500 nM for 1, 3, 6, 12 or 24 h, with untreated control samples also prepared at each time point. Total RNA was then extracted using Qiagen RNeasy Plant Mini kit, according to manufacturer's instructions. RNA aliquots were checked for integrity and quantity on an Agilent 2100 Bioanalyzer using an RNA 6000 nano chip kit (Agilent). RNA‐seq libraries were prepared using 5 μg of total RNA with True Seq RNA Sample Preparation kit from Illumina (Agilent) according to manufacturer's instructions. One hundred base pair, paired‐end reads were sequenced from mRNA samples on the Illumina HiSeq 2500 (Illumina, Inc.) using ScriptSeq reagents (Illumina, Inc.). Reads were filtered using the fastq‐mcf program from the ea‐utils package (http://code.google.com/p/ea‐utils/) applying −x 0.01, −q 20, −p 10, and −u. Reads were mapped to the *Magnaporthe oryzae* 70‐15 reference genome version 8 (Dean *et al*., 2005) using the TopHat2 package (Kim *et al*., 2013), which takes account of splice sites. Counts of reads mapping to each gene in the *M. oryzae* genome were generated using the htseq‐count function of the HTSeq package (Anders *et al*., 2015). Relative gene expression was quantified and differentially expressed genes were identified using DESeq (Anders and Huber, 2010). All RNA‐seq data from this study have been uploaded to GEO (Gene Expression Omnibus) at NCBI, under accession number GSE70308.

## Supporting information

Supporting information
