# Supplementary material for: Protein kinase C is essential for viability of the rice blast fungus M agnaporthe oryzae
Source: Mol Microbiol. 2015 Aug 18;98(3):403–19. doi: 10.1111/mmi.13132 (PMC4791171; doi:10.1111/mmi.13132)
Supplement: Supplementary file 1 — Supporting information [file MMI-98-403-s001.zip › MMI_13132_supp-0001-Figure_S1.pdf]

```
                                            HR1
                            *        20         *        40         *        60
M.oryzae       : ---MDDRIQDIYKKIEREKALINAANLMR----QQTNNDAVRSKLDTQMREGRRNLEFFE :  53
N.crassa       : -MNDEDKVHDISKKIEREKALINAAQAMR----QQTNNEQVRSKLDTQMREGRRNLEFFE :  55
A.nidulans     : -MDGDELIASVYRKIEREKALIAAASNMR----QSTDNPLVQQRVDANIRDGRKNIAYLE :  55
C.neoformans   : MASNDPKAKVILQSIATEKRNIEGARAVIRAFEASSKNETVIQQAQNEIRTATMSIKFLE :  60
S.cerevisiae   : -MSFSQLEQNIKKKIAVEENIIRGASALK----KKTSNVMVIQKCNTNIREARQNLEYLE :  55
H.sapiens_ep   : ------------------------------------------------------------ :   -

                            *        80         *       100         *       120
M.oryzae       : GTLREMQMRSMGQGMDNLSIGGSTLAASASSSSRPRSAVEEDGPMPPPKDGGYGAGGDGY : 113
N.crassa       : EKLRELQMRRLGHGVDNMSLGASPMSGSHRQSVDDFEGYG--APSPPPKE-------DVR : 106
A.nidulans     : EKMKELQLRQMER------ESGSPTDKRLPPNPED-------GPAPPPKD------PLGY :  96
C.neoformans   : DELAKLQVGGSGG--------ASGTPGRGEPSGGT-------GAGVPGKQ--------GP :  97
S.cerevisiae   : DSLKKLRLKTAQQ------------SQGENGSED-------NERFNSKE----------Y :  86
H.sapiens_ep   : ------------------------------------------------------------ :   -

                            *       140         *       160         *       180
M.oryzae       : GQTQYSQIGEHGDLMPPRGPFANQGPGSHIPKTR----PNFTKLDLIKYDTPYLGPRIQL : 169
N.crassa       : GHSSHQSQGS-GPLMPASAPYPGGPPDSNVPRAR----PNYTRLDLIKFDTPHLGPRIQL : 161
A.nidulans     : GDPTQHPQPG-AGSMPSGAPFKDPRPFAPVPKAR----PNYTKLDLIKYDTPYLGPKIQL : 151
C.neoformans   : QGVVASSPGGRGMAGDRERPLPPPPPGAEQDSAKKPETKNYSQLDLLRYDAPLTGAKITR : 157
S.cerevisiae   : GFLSTKSPNE----------------------------HIFSRLDLVKYDCPSLAQRIQY : 118
H.sapiens_ep   : ---------------------------------------------------------MVV :   3

                                            HR1
                            *       200         *       220         *       240
M.oryzae       : MLSQIQFKLNVEEQYLKGIEKMVQLYQMEGDKKSRADAAARRVESKQKIVLLKQALRRYE : 229
N.crassa       : MLSQIQFKLNVEEQYLKGIEKMVQLYQMEGDKKSKLDAAAKRVESKQKIVLLKQALKRYE : 221
A.nidulans     : MLSQLEFKLSVEKQYKAGIEKMVRLYQDEGDRKSRTDAEGRRIESNQKIQLLKQALKRYE : 211
C.neoformans   : MLNQLQFKLQVEEQYKTGIEKMAQAYRAEGDKRLKSETDAKKSESEGKIQLLRKAKKRYE : 217
S.cerevisiae   : MLQQLEFKLQVEKQYQEANTKLTKLYQIDGDQRSSSAAEGGAMESKYRIQMLNKALKKYQ : 178
H.sapiens_ep   : FNGLLKIKICEAVSLKPTAWSLRHAVG--------------------------------- :  30

                            *       260         *       280         *       300
M.oryzae       : ELHIDIDSGDSPDD---DSINMPNLRKPLTGQLSIRVIAIKDVDHAPTGRFARG--PDTF : 284
N.crassa       : ELHIDID-VDGPDD---DSINLPALRKPLSGTLSIRILAVKDVDHAPLGRFARS--PETF : 275
A.nidulans     : DLHVDIESTDTPDD---ESLSAPNLRKPLTGLLTMRIQAVQDVDHATSSRFSRG--PETF : 266
C.neoformans   : SLAKFGGAVEDDEDLMSDGKRKEALRKPISGKLVISLRSARDLNHRALPRRSSKTYSETT : 277
S.cerevisiae   : AINVDFDQFKHQPNDIMDNQQPKFRRKQLTGVLTIGITAARDVDHIQSPMFARK--PESY : 236
H.sapiens_ep   : ---------------------------PRPQTFLLDPYIALNVDDSRIGQTATK------ :  57

                                            C2
                            *       320         *       340         *       360
M.oryzae       : VAIKVEDNIAARTRVSRTDRWENEYHNVEVDKANEIELTVYDKPGE-HSIPIGMLWVRIS : 343
N.crassa       : IAVKAEDIVVARTKPSRNDKWEAEFHTFPVDKTNEIEFTVYDKPAE-HPVPIAMLWVRIS : 334
A.nidulans     : VIVKVEDTIKARTKATRSDRWQDETFNLDIDKANEVELTVYDKAGD-RPTPIGMLWLRIS : 325
C.neoformans   : VVIKVEGNERAVSHPSRNDKWHEDFH-IPVEKANEVEITIYDTVAPGDSAPIGMLWLRVS : 336
S.cerevisiae   : VTIKIDDTIKARTKPSRNDRWSEDFQ-IPVEKGNEIEITVYDKVND-SLIPVAIMWLLLS : 294
H.sapiens_ep   : -------------QKTNSPAWHDEFVTDVCN-GRKIELAVFHDAPIGYDDFVANCTIQFE : 103

                            *       380         *       400         *       420
M.oryzae       : DIAEELRRKKIEAETNSLGWVSADRMGDVNPAARVPPPQFPMNTQQQQPGGP-VSPGIQG : 402
N.crassa       : DIVEELRRKKIEAEMTSAGWVSADRVG-----SRAPPPQFPMGAQSPQFAAPPTSPGSQ- : 388
A.nidulans     : DIAEEMRRKKIETEINASGWVSADKMG-PGIGPGQTTPAAPVGSSQYGASAGYSNPGPEG : 384
C.neoformans   : DLVEALRRQKVGIEGQGAGWVTAATAA---TMGPRSSGNAPDSVTLHSAGTLRGKPDAEG : 393
S.cerevisiae   : DIAEEIRKKKAGQTNEQQGWVNASNIN---GGSSLASEEGSTLTSTYSNSAIQSTSAKNV : 351
H.sapiens_ep   : ELLQNGSR---------------------------------------------------- : 111

                            *       440         *       460         *       480
M.oryzae       : QQQGYGQPGDPQQPGQQGQMGQVQEPITGWFNLEPTGSIQLSLNFFKQ--SKDRRPVDLG : 460
N.crassa       : -EQNTMIPPQAPPPSQ-----VVSQPVDGWFNLEPYGQIHLSFNFIKG--ARPQG-MDR- : 438
A.nidulans     : QTGATGGP----------------LMIDAWFALEPVGRIHISMSFAKH--SKDRRPFDIG : 426
C.neoformans   : -----KGP----------------DGIDGWWSVEPAGAISLRMDFVKDTVAGARRPYEA- : 431
S.cerevisiae   : QGENTSTS---------------QISTNSWFVLEPSGQILLTLGFHKSS-QIERKQLMGG : 395
H.sapiens_ep   : -------------------------HFEDWIDLEPEGRVYVIIDLSGSSGEAPKDNEERV : 146

                   Pseudosubstrate                        C1A
                            *       500         *       520         *       540
M.oryzae       : LGRKGAIRQR----KEEVHEMYGHKFVERQFYNIMRCALCGDFLKGGS---GMQCEDCKY : 513
N.crassa       : LGRKGAVRQR----KEEVHEMYGHKFVQKQFYNIMRCALCGDFLKYSA---GMQCEDCKY : 491
A.nidulans     : LNRQGAVRQR----KEEVHEKQGHKFVTQQFYNIMRCALCGDFLKYAA---GMQCSDCKY : 479
C.neoformans   : LGRQGAVRMR----KGDVYEMNGHKFVQRQFYQPIMCALCQEFLLTGE---GYQCEDCRY : 484
S.cerevisiae   : LHRHGAIINR----KEEIFEQHGHHFVQKSFYNIMCCAYCGDFLRYT----GFQCQDCKF : 447
H.sapiens_ep   : FRERMRPRKRQGAVRRRVHQVNGHKFMATYLRQPTYCSHCRDFIWGVIGKQGYQCQVCTC : 206
```

```
                                                                          C1B
                             *       560         *       580         *       600
M.oryzae       : TCHIKCYTSVVTKCISKSNAET--DPDEEKINHRIPHRFVAFSNLTANWCCHCGYLLPFG :   571
N.crassa       : TCHTKCYTSVVTKCISKSNAET--DPDEEKINHRIPHRFIPFSNLTANWCCHCGYMLPIG :   549
A.nidulans     : TCHRKCYPKVVTKCISKANYET--DPDEEKINHRIPHRFEGFSNISANWCCHCGYMLPFG :   537
C.neoformans   : ACHKKCYPKVVTKCISKSNADG--EGDEEKINHRIPHRFTPYTNMSANWCCHCGYMLPFG :   542
S.cerevisiae   : LCHKKCYTNVVTKCIAKTSTDT--DPDEAKLNHRIPHRFLPTSNRGTKWCCHCGYILPWG :   505
H.sapiens_ep   : VVHKRCHELIITKCAGLKKQETPDQVGSQRFSVNMPHKFGIHNYKVPTFCDHCG-SLLWG :   265

                             *       620         *       640         *       660
M.oryzae       : -KKNCRKCS---ECQMAAHAGCVHLVPDFCGMSMAVANQILEGIRSQKQ-RQQKASS--- :   623
N.crassa       : SKKNSRKCS---ECALTAHAQCVHLVPDFCGMSMAVANQILEGMRTQKKTHKDKASS--- :   603
A.nidulans     : -RKNAKRCS---ECGLTCHAHCTHLVPDFCGMSMEAANQILETLIRHKNHNKSPSVSS-- :   591
C.neoformans   : -RKNAVKCSDITECALTCHQTCSHLVPDFCGMTMEMANLLLKNLRDIKTTQHRKPVP--- :   598
S.cerevisiae   : -RHKVRKCS---ECGIMCHAQCAHLVPDFCGMSMEMANKILKTIQDTKRNQEKKKRTVPS :   561
H.sapiens_ep   : LLRQGLQCK---VCKMNVHRRCETNVAPNCGVDARGIAKVLADLGVTPDKITNSGQRR-- :   320

                             *       680         *       700         *       720
M.oryzae       : --MSDRTLRSGKMSPPGSGHASSASFSQGMGSSYGQASPEATEAAKFMYSSQTSPQRITS :   681
N.crassa       : --MSERTLRPGSKTSISSGSIAQASTYSGSTAYTSIASPEATEAAKLMYS-QTTP-RPGG :   659
A.nidulans     : -GLSGKTLRPGGSQDAGHAYPKPVESPSSYGQRPPSAEAVSAAATSYIPPPQSPTSQRQP :   650
C.neoformans   : -----STSTSSSVSTLPSYHSQESRSHPVQSAALQQPAPSSRPPAGALPSTDQRPIQPQV :   653
S.cerevisiae   : AQLGSSIGTANGSDLSPSKLAERANAPLPPQPRKHDKTPSPQKVGRDSPTKQHDPIIDKK :   621
H.sapiens_ep   : -----KKLIAGAESPQPASGSSPSEEDRSKSAPTSPCDQEIKELENNIRKALSFDNRGEE :   375

                             *       740         *       760         *       780
M.oryzae       : PDRTSSS-----------------SQAAAAATAAMTGLPGAMSSQPGRQGQQPITDYGSA :   724
N.crassa       : PDRTSTS-----------------STTASAAAAAAMAP--KHSSQPSQAG--SIPDFGGS :   698
A.nidulans     : LPPRTSS-----------------SSPAAAAAAVATGR---MPGRPVQP----------- :   679
C.neoformans   : QNQQTGA-----------------YDNLRPAGGRAMPQ-----LPPVAV----------- :   680
S.cerevisiae   : ISLQTHGREKLNKFIDENEAYLNFTEGAQQTAEFSSPEKTLDPTSNRRSLGLTDLSIEHS :   681
H.sapiens_ep   : HRAASSP----------------------------------------------------- :   382

                             *       800         *       820         *       840
M.oryzae       : SGGRYGSYGPHDDPYAQPQQSPPPQQQAAYGQPQQRKYNPADYANISGGYGSQPQMAQQP :   784
N.crassa       : PG--YGRPDSRDDEYS-------AQQQQGYGSPQQRKYNPADYANIDAYSSPQARPQQQQ :   749
A.nidulans     : --------------------------------PAHAHYDPAAYASYQQGAMQQGYPPAQA :   707
C.neoformans   : --------------------------------PSNKISFEGDRLQEPHGQIPPVMPAAQP :   708
S.cerevisiae   : QTWESKDDLMRDELELWKAQREEMELEIKQDSGEIQEDLEVDHIDLETKQKLDWENKNDF :   741
H.sapiens_ep   : ---------------------------------DGQLMSPGENGEVRQGQAK-------- :   401

                             *       860         *       880         *       900
M.oryzae       : QQARPQQQQQQPLYSPQQHASQAQQPLSPVKQQHQEQQIISPTAGTVIPTSAKRPLPS-- :   842
N.crassa       : QQQQQTPQQVSPMY--QQNPQTPISKPQPVAPSYDNQVVPSASG---VPVPTKKPLPS-- :   802
A.nidulans     : MQKVNAPAQY------GMHAPPPPQQQQQMQAMQQQ-------------VAAKEDIPQ-- :   747
C.neoformans   : QPQTLPPSAQS-----VQSPVKPQYVPQPAVQQQVAQR-----------LPPQQPLMA-- :   750
S.cerevisiae   : READLTIDSTHTNPFRDMNSETFQIEQDHASKEVLQETVSLAPTSTHPSRTTDQQSPQKS :   801
H.sapiens_ep   : ------------------------------------------------------------ :     -

                             *       920         *       940         *       960
M.oryzae       : --------ATDPGTGQRIGLDHFNFLAVLGKGNFGKVMLAESKKTRKLYAIKVLKKEFII :   894
N.crassa       : --------ATDPGTGMRIGLDHFNFLAVLGKGNFGKVMLAETKKSRKLYAIKVLKKEFII :   854
A.nidulans     : --------QPK----VRIGLDHFNFLAVLGKGNFGKVMLAETKATKKLYAIKVLKKEFII :   795
C.neoformans   : --------RKR-----KVGLDDFNFLAVLGKGNFGKVMLAEEKTSSNLYAIKVLKKEFII :   797
S.cerevisiae   : QTSTSAKHKKRAAKRRKVSLDNFVLLKVLGKGNFGKVILSKSKNTDRLCAIKVLKKDNII :   861
H.sapiens_ep   : ----------------RLGLDEFNFIKVLGKGSFGKVMLAELKGKDEVYAVKVLKKDVIL :   445

                             *       980         *      1000         *      1020
M.oryzae       : ENDEVESIRSEKRVFLIANRERHPFLTNLHACFQTETRVYFVEEYISGGDLMLHIQRGQ- :   953
N.crassa       : ENDEVESIRSEKRVFLIANRERHPFLTNLHACFQTETRVYFVMEYISGGDLMLHIQRGM- :   913
A.nidulans     : ENDEVESTKSEKRVFLIANKERHPFLLNLHACFQTETRVYFVMEYISGGDLMLHIQRGQ- :   854
C.neoformans   : ENDEVESTQSEKRVFLAAAQERHPFLLGLHSCFQTETRVYFVMEYISGGDLMLHIQKKQ- :   856
S.cerevisiae   : QNHDIESARAEKKVFLLATKTKHPFLTNLYCSFQTENRIYFAMEFIGGGDLMWHVQNQR- :   920
H.sapiens_ep   : QDDDVDCTMTEKRILALAR--KHPYLTQLYCCFQTKDRLFFVMEYVNGGDLMFQIQRSRK :   503

                             *      1040         *      1060         *      1080
M.oryzae       : FGTRRAQFYAAEVCLALKYFHENGVIYRDLKLDNILLTLDGHIKIADYGLCKEDMWYGST :  1013
N.crassa       : FGTKRAQFYAAEVCLALKYFHENGVIYRDLKLDNILLTLDGHIKIADYGLCKEDMWYGST :   973
A.nidulans     : FGLKRAQFYAAEVLLALKYFHENGVIYRDLKLDNILLTLDGHIKIGDYGLCKENMWYGST :   914
C.neoformans   : FTLRQAKFYACEVLLALQYFHSKGIIYRDLKLDNILLTLDGHVKVADYGLCKEEMWFGKT :   916
S.cerevisiae   : LSVRRAKFYAAEVLLALKYFHDNGVIYRDLKLENILLTPEGHIKIADYGLCKDEMWYGNR :   980
H.sapiens_ep   : FDEPRSRFYAAEVTSALMFLHQHGVIYRDLKLDNILLDAEGHCKLADFGMCKEGILNGVT :   563
```

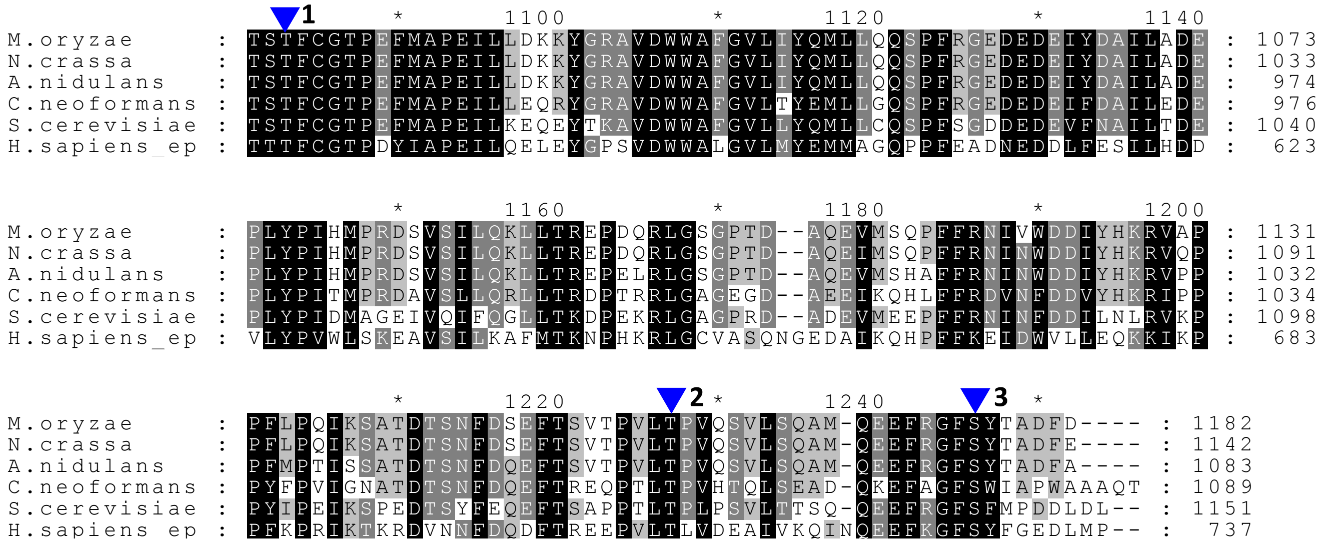

Figure S1. Alignment of the predicted amino acid sequence of Pkc1 with fungal PKC-encoding genes.

The predicted amino acid sequence of *M. oryzae* Pkc1 was aligned with *N. crassa* Pkc1 (XP_962251), *A. nidulans* Pkc1 (XP_657710), *C. neoformans* Pkc1 (XP_569656), *S. cerevisiae* Pkc1 (EDN64518) and *H. sapiens* PKC-epsilon type (NP_005391). Sequences were aligned using the program ClustalW (Thompson *et al*., 1994).). Identical amino acids are highlighted on a black background and residues with a grey background are identical for at least 50% of listed proteins. The conserved PKC domains are highlighted. ▼ Indicates the 3 conserved phosphorylation sites: 1) in the activation loop, 2) in the turn motif and 3) in the hydrophobic motif.
