# Supplementary material for: Protein kinase C is essential for viability of the rice blast fungus M agnaporthe oryzae
Source: Mol Microbiol. 2015 Aug 18;98(3):403–19. doi: 10.1111/mmi.13132 (PMC4791171; doi:10.1111/mmi.13132)
Supplement: Supplementary file 1 — Supporting information [file MMI-98-403-s001.zip › MMI_13132_supp-00011-TableS3.docx]

**Table S3. Primers used in this study**

| Primer | Gene | Flank | Sequence (5' – 3') |
| --- | --- | --- | --- |
| Mgg08689.6(1) | PKC1 | Left | GTTACCTGCTGTCTGTCAAAAG |
| Mgg08689.6(2) | PKC1 | Left | GTCGTGACTGGGAAAACCCTGGCGTTTTGGAAGCCTGTCTCG |
| Mgg08689.6(3) | PKC1 | Right | TCCTGTGTGAAATTGTTATCCGCTGTCCTGACGCCAGTCCAAT |
| Mgg08689.6(4) | PKC1 | Right | GATGGATGAGGTCAAGCAGGAG |
| Mgg14873.6(1) | MDL1 | Left | CATACGCATTCACAGCTTCC |
| Mgg14873.6(2) | MDL1 | Left | GTCGTGACTGGGAAAACCCTGGCGCCTTGGTATTGAGTCATTCTGG |
| Mgg14873.6(3) | MDL1 | Right | TCCTGTGTGAAATTGTTATCCGCTCGGTAGTCCTTTCCCATTT |
| Mgg14873.6(4) | MDL1 | Right | AACGAGAAACATACCACTTGATG |
| Mgg12357.6(1) | MDL2 | Left | AGTCAACACTGCAAAGCCTACG |
| Mgg12357.6(2) | MDL2 | Left | GTCGTGACTGGGAAAACCCTGGCGTTCAGTGACGGTCGGTAAGTAAC |
| Mgg12357.6(3) | MDL2 | Right | TCCTGTGTGAAATTGTTATCCGCTTTGTGGAGCGTGGTGAGGA |
| Mgg12357.6(4) | MDL2 | Right | AGATGGCTGCCGCTGGTA |
| HY split | HYG |  | GGATGCCTCCGCTCGAAGTA |
| YG split | HYG |  | CGTTGCAAGACCTGCCTGAA |
| BA split | BAR |  | GGACTTCAGCAGGTGGGTGTAGAG |
| AR split | BAR |  | GCAGACAGGAACGAGGACATTA |
| IL split | ILVI |  | TCTGGTTGTATTCTCAGGAC |
| LV split | ILVI |  | CATACCAAGCATGTGCAGTG |
| M13F |  |  | CGCCAGGGTTTTCCCAGTCACGAC |
| M13R |  |  | AGCGGATAACAATTTCACACAGGA |

The reverse complement of M13 forward and reverse sequences used for fusion PCR are shown underlined.
