# Supplementary material for: Protein kinase C is essential for viability of the rice blast fungus M agnaporthe oryzae
Source: Mol Microbiol. 2015 Aug 18;98(3):403–19. doi: 10.1111/mmi.13132 (PMC4791171; doi:10.1111/mmi.13132)
Supplement: Supplementary file 1 — Supporting information [file MMI-98-403-s001.zip › MMI_13132_supp-0002-Figure_S2.pdf]

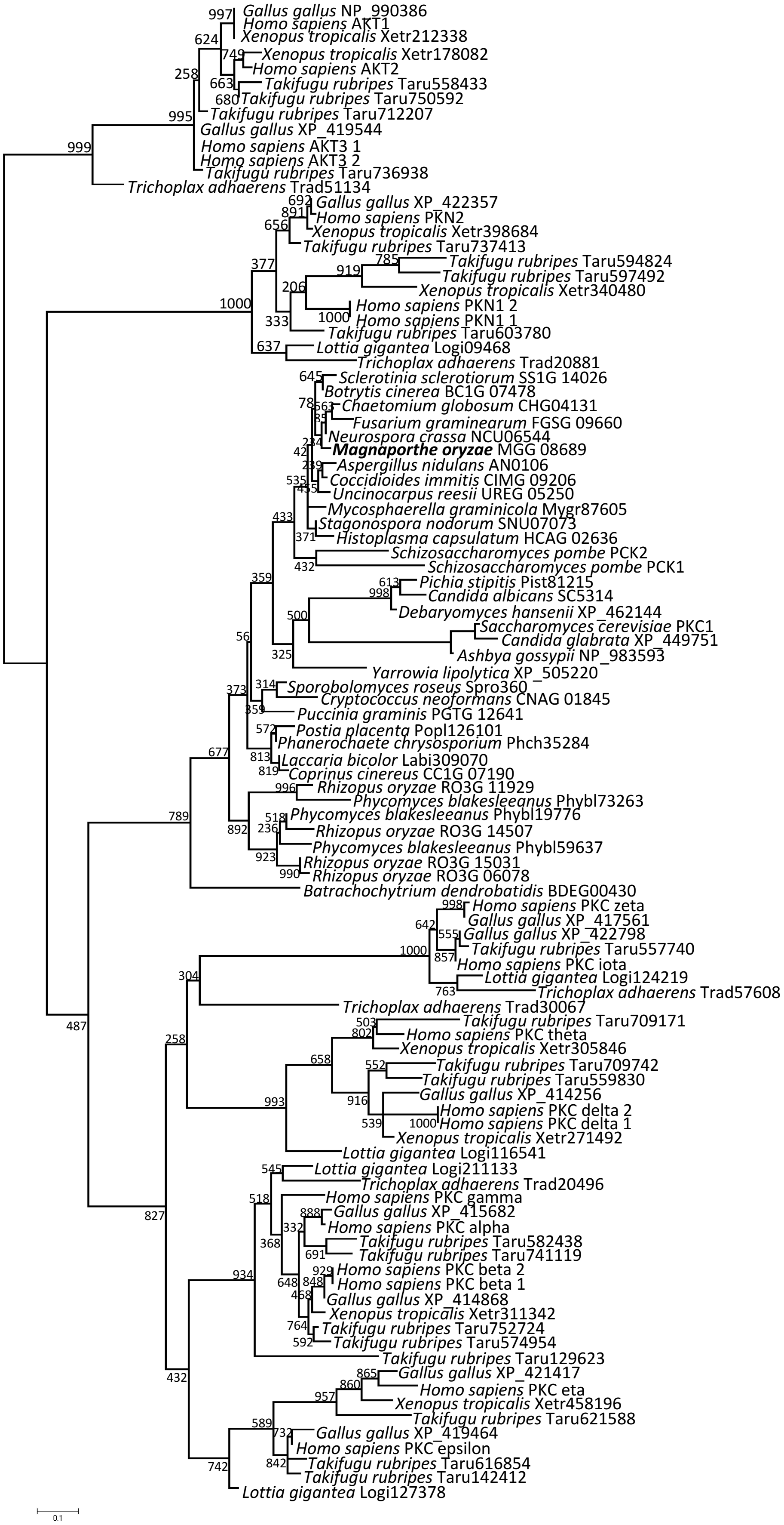

Figure S2. Phylogenetic analysis of PKC amino acid sequences from a range of organisms

A maximum likelihood tree (Felsenstein, 1981) was constructed from amino acid sequences of a broad cross-section of *PKC*-encoding genes from diverse eukaryotes. Tree topology was tested by 1000 bootstrap re-sampling of the data. *PKC1* is part of a clade of fungal-specific *PKC*-encoding genes with strong bootstrap support, within which the ascomycete-specific PKCs forms a single clade.
