# Supplementary material for: Protein kinase C is essential for viability of the rice blast fungus M agnaporthe oryzae
Source: Mol Microbiol. 2015 Aug 18;98(3):403–19. doi: 10.1111/mmi.13132 (PMC4791171; doi:10.1111/mmi.13132)
Supplement: Supplementary file 1 — Supporting information [file MMI-98-403-s001.zip › MMI_13132_supp-0003-Figure_S3.pdf]

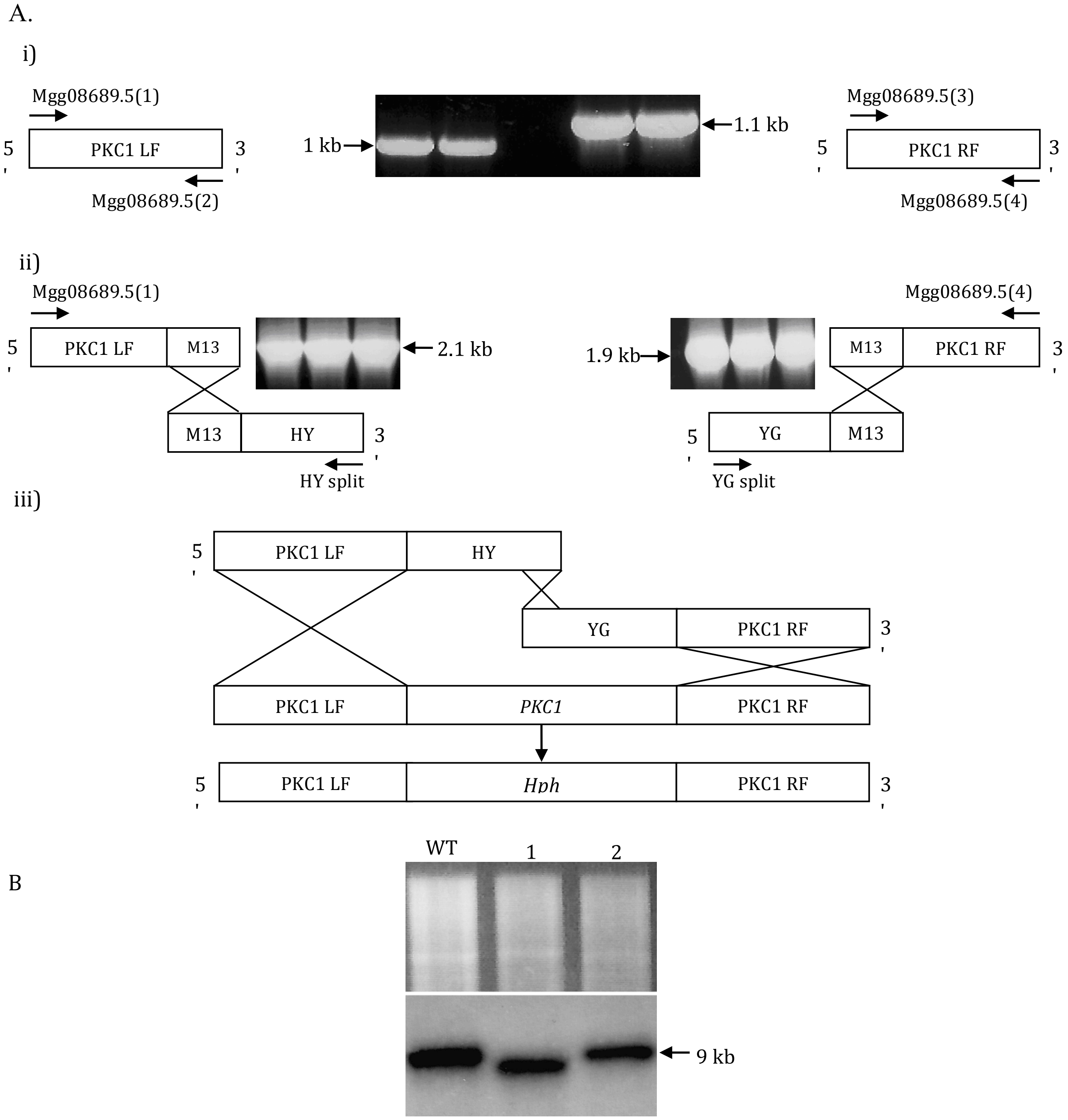

Figure S3. A schematic representation of the attempted targeted deletion of *PKC1* by the split-marker deletion method

A. i) PCR amplification of the 5' and 3' flanks of the *PKC1* open reading frame (ORF) was carried out with primers for the 5' and 3' inner flanks designed to include an extension complementary to the tail of a fragment of the selectable marker, the hygromycin phosphotransferase cassette (HYG). ii) In a second round of PCR, the flanks of *PKC1* were fused with overlapping marker fragments, HY and YG, of the hygromycin phosphotransferase cassette. iii) Homologous recombination between the flanking regions and chromosomal DNA and between the overlapping regions of the hygromycin phosphotransferase cassette should result in a targeted deletion.

b) Genomic DNA from the two hygromycin resistant transformants was restriction digested with *Xba* I, fractionated by gel electrophoresis and transferred to Hybond-N. The Southern blot was subsequently probed with a 1 kb fragment from the *PKC1* locus. The presence of a hybridising fragment in both transformants shows that neither contained a correctly targeted deletion.
