# Supplementary material for: Protein kinase C is essential for viability of the rice blast fungus M agnaporthe oryzae
Source: Mol Microbiol. 2015 Aug 18;98(3):403–19. doi: 10.1111/mmi.13132 (PMC4791171; doi:10.1111/mmi.13132)
Supplement: Supplementary file 1 — Supporting information [file MMI-98-403-s001.zip › MMI_13132_supp-0004-Figure_S4.docx]

**Figure S4.**  Heatmap showing Euclidean distances between RNA-seq samples, as calculated from a variance-stabilising transformation of the total count data. The darker the colour, the closer the two datasets are together. RNA-Seq analysis was performed on M. oryzae *pkc1^AS^* mutant following selective kinase inhibition with 500 nM 1NA-PP1. T3, T6, T12, T24 are time-points following 1NA-PP1 exposure. POS = 1NA-PP1 treatment, NEG = untreated control experiment.
