# Supplementary material for: Protein kinase C is essential for viability of the rice blast fungus M agnaporthe oryzae
Source: Mol Microbiol. 2015 Aug 18;98(3):403–19. doi: 10.1111/mmi.13132 (PMC4791171; doi:10.1111/mmi.13132)
Supplement: Supplementary file 1 — Supporting information [file MMI-98-403-s001.zip › MMI_13132_supp-0006-Figure_S6.docx]

**
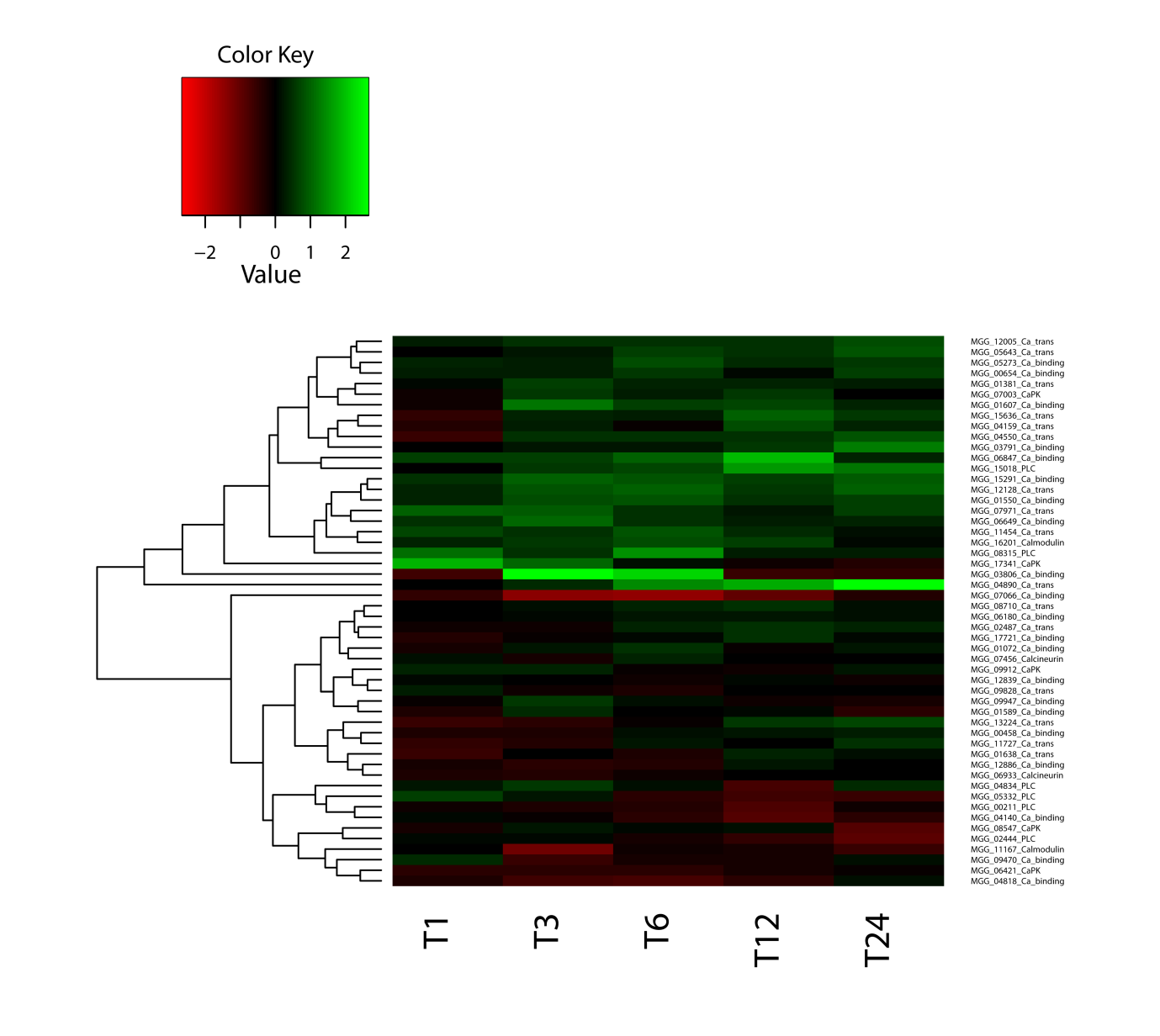
**

**Figure S6.** Heatmap showing log_2_ expression ratio of genes predicted to be involved in calcium signalling, following selective protein kinase C inhibition of the *pkc1*^as^ mutant of *M. oryzae* with 1NA-PP1. Key: Ca_trans = calcium transporter, Ca_binding = calcium / calmodulin binding protein, CaPK = calmodulin / calcium dependent protein kinase, PLC = phospholipase C**.** Highly significant differences include MGG_04890 - vacuolar Ca2+ ATPase - up-regulated at 12h, 24h and MGG_07066 - calcium-binding mitochondrial carrier protein – down-regulated at 6h
