# Supplementary material for: Protein kinase C is essential for viability of the rice blast fungus M agnaporthe oryzae
Source: Mol Microbiol. 2015 Aug 18;98(3):403–19. doi: 10.1111/mmi.13132 (PMC4791171; doi:10.1111/mmi.13132)
Supplement: Supplementary file 1 — Supporting information [file MMI-98-403-s001.zip › MMI_13132_supp-0008-Supplemental.docx]

**Supplemental Experimental Procedures**

**Generation of the *Δpkc1* gene replacement vector**

Targeted gene replacement of *PKC1* was attempted using a PCR-based, split-marker deletion method (Yu *et al.*, 2004), as illustrated in Figure S3a. Gene-specific primers were designed to allow gene coding sequence amplification of 1 kb fragments fom the 5' and 3' flanks of the *PKC1* open reading frame (ORF). The primers for the 5' and 3' inner flanks were each designed to include an extension that was complementary to the ends of a fragment of the hygromycin phosphotransferase gene cassette (*Hph*), to facilitate the fusion of the *PKC1* flanking sequence and the selectable marker gene. In a second round of PCR, two constructs were formed, each containing a 1 kb flank of the *PKC1* gene and an overlapping region of the *Hph* cassette. This construct was then transformed into the Δ*ku*70 strain of *M. oryzae*, which lacks the non-homologous DNA end-joining pathway (Krappmann *et al.*, 2006), thereby ensuring that DNA could only be integrated by homologous recombination (Kershaw & Talbot, 2009). Putative transformants were selected based on their resistance to hygromycin B (200 μg ml^-1^). Failure to recover any Δ*pkc1* transformants from five independent experiments is consistent with PKC1 serving an essential function in *M. oryzae*.

### **Chemical synthesis of PP1 and analogues**

The three protein kinase inhibitors **1a**-**c** (Bishop *et al*., 1999, 2000) (Figure 1), all based on a central pyrazolo-pyrimidine structural motif, were prepared using minor modifications to a previously reported methodology (Hanefeld *et al.*, 1996) (Scheme 1).

**Figure 1 PP1 and two structural analogues**

**Scheme 1 Outline synthetic route to 1a-c**

Where appropriate, sufficient basic characterisation was carried out on all compounds prepared to confirm structural assignments with those already reported in the literature.

For general experimental details, see Wood *et al* (2010).

Firstly, malononitrile **2** was acylated with the acid chloride **3** corresponding to the desired C-3 side-chain substituent R. 1-Naphthaleneacetyl chloride required for the preparation of **1c**, is not commercially available and this was prepared from 1-naphthalenacetic acid using thionyl chloride, prior to carrying out the acylation. (Note: the acylated derivatives **4a**-**c** were found to exist predominantly as their enol tautomers.)

**2-(Hydroxy(4-methylphenyl)methylene)malononitrile 4a**

4-Methylbenzoyl chloride **3a** (6.62 cm^3^, 50 mmol) was added dropwise to a stirred mixture of malononitrile **2** (3.305 g, 50 mmol) and sodium hydride (60% dispersion in mineral oil, 4.00 g, 100 mmol) in tetrahydrofuran (50 cm^3^), under a nitrogen atmosphere, maintaining the temperature between 5 and 10 °C with the aid of an ice-salt bath. After allowing the stirred reaction mixture to attain room temperature, hydrochloric acid (1 M, 125 cm^3^) was added and the mixture thus obtained was extracted with ethyl acetate (3 x 50 cm^3^). The combined extracts were dried (magnesium sulfate), filtered and evaporated *in vacuo* to give an off-white solid. Recrystallisation from ethyl acetate gave the *title compound* **4a** (2.90 g, 32%) as an off-white, crystalline solid. m.p. 192-193 °C;* *_H_ (300 MHz; DMSO-d_6_) 2.36 (3H, s, C*H*_3_), 5.26 and 7.28, 7.54 (2 x 2H, AA'BB', *J* = 7.5, arylC*H*);* *_C_ (75 MHz; DMSO-d_6_) 21.3 (*C*H_3_), 53.3 (*C*(CN)_2_), 118.1 (*C*≡N), 119.6 (*C*≡N), 128.0, 128.9 (aryl*C*H), 133.5 (aryl*C*C=O), 141.5 (aryl*C*CH_3_) and 186.7 (*C*(OH)).

**2-(Hydroxy(naphthalen-1-yl)methylene)malononitrile 4b**

1-Naphthoyl chloride **3b** (3.95 cm^3^, 26.2 mmol) was added dropwise to a stirred mixture of malononitrile **2** (2.19 g, 33.2 mmol) and sodium hydride (60% dispersion in mineral oil, 2.65 g, 66.3 mmol) in tetrahydrofuran (35 cm^3^), under a nitrogen atmosphere, maintaining the temperature between 5 and 10 °C with the aid of an ice-salt bath. After allowing the stirred reaction mixture to attain room temperature, hydrochloric acid (1 M, 80 cm^3^) was added and the mixture thus obtained was extracted with ethyl acetate (3 x 50 cm^3^). The combined extracts were dried (magnesium sulfate), filtered and evaporated *in vacuo* to give a white, crystalline solid suspended in a red-brown oil. Trituration with hexane gave the *title compound* **4b** (3.96 g, 69%) as a white, crystalline solid, which was used without further purification. m.p. 131-135 °C;* *_H_ (300 MHz; DMSO-d_6_) 7.54-7.66 (4H, complex, arylC*H*), 7.93 (1H, m, arylC*H*) and 7.98-8.08 (2H, complex, arylC*H*);* *_C_ (75 MHz; DMSO-d_6_) 57.0 (*C*(CN)­_2_), 117.0 (*C*≡N), 118.3 (*C*≡N), 125.0, 125.3, 125.8, 126.7, 127.4, 128.6 (aryl*C*H), 129.5 (aryl*C*), 130.4 (aryl*C*H), 133.2, 134.5 (aryl*C*) and 187.9 (*C*(OH)).

**2-(1-Hydroxy-2-(naphthalen-1-yl)ethylidene)malononitrile 4c**

Thionyl chloride (10.0 cm^3^, 137 mmol) was added to 1-napthaleneacetic acid (10.00 g, 54 mmol) and the resulting mixture was heated under reflux for 3.5 h. The excess thionyl chloride was removed by conventional vacuum distillation (oil bath temperature 80 °C, 15 mmHg), followed by azeotropic distillation with hexane (4 x 30 cm^3^) on a rotary evaporator and the resulting crude product, obtained as a dark orange/brown oil, was used in the next step without further purification/characterisation. The crude acid chloride (assumed to be **3c** 10.99 g, 54 mmol) was added dropwise to a stirred mixture of malononitrile (3.57 g, 54 mmol) and sodium hydride (60% dispersion in mineral oil, 4.32 g, 108 mmol) in tetrahydrofuran (50 cm^3^), under a nitrogen atmosphere, maintaining the temperature between 5 and 10 °C with the aid of an ice-salt bath. After allowing the stirred reaction mixture to attain room temperature, hydrochloric acid (1 M, 150 cm^3^) was added and the mixture thus obtained was extracted with ethyl acetate (3 x 50 cm^3^). The combined extracts were dried (magnesium sulfate), filtered and evaporated *in vacuo* to give an impure product, assumed to be the *title compound* **4c** (11.23 g) as an orange foam, which was used without any further purification or characterisation.

The three products **4a**-**c** were then converted into their corresponding enol ethers **5a**-**c** by methylation with dimethyl sulfate in the presence of sodium hydrogen carbonate.

**2-(Methoxy(4-methylphenyl)methylene)malononitrile 5a**

2-(Hydroxy(4-methylphenyl)methylene)malononitrile **4a** (2.76 g, 15.0 mmol) and dimethyl sulfate (10.00 cm^3^, 105.7 mmol) were added to a stirred mixture of sodium hydrogen carbonate (10.0 g, 120.0 mmol), 1,4-dioxane (24 cm^3^) and water (4 cm^3^). After stirring for 2.5 h at 80-90 °C, water (120 cm^3^) was added to the cooled reaction mixture and the resulting mixture was extracted with *tert*-butyl methyl ether (4 x 60 cm^3^). The combined extracts were dried (sodium sulfate), filtered and evaporated to give an off-white, crystalline solid. Recrystallisation from methanol gave the *title compound* **5a** (1.20 g, 40%) as a white, crystalline solid. *δ*_H_ (300 MHz; DMSO-d_6_) 2.43 (3H, s, C*H*_3_Ar), 3.90 (3H, s, C*H*_3_O) and 7.46, 7.59 (2 x 2H, AA'BB', *J* = 9.0) arylC*H*);* *_C_ (75 MHz; DMSO-d_6_) 21.4 (*C*H_3_Ar), 61.8 (*C*H_3_O), 65.4 (*C*(CN)_2_), 112.6 (*C*≡N), 114.1 (*C*≡N), 125.2 (aryl*C*), 129.0 (aryl*C*H), 129.9 (aryl*C*H), 143.5 (aryl*C*) and 186.9 (CH_3_O*C*).

**2-(Methoxy(naphthalen-1-yl)methylene)malononitrile 5b**

2-(Hydroxy(naphthalen-1-yl)methylene)malononitrile **4b** (3.50 g, 15.9 mmol) and dimethyl sulfate (10.6 cm^3^, 112.0 mmol) were added to a stirred mixture of sodium hydrogen carbonate (10.6 g, 126.2 mmol), 1,4-dioxane (25 cm^3^) and water (4.25 cm^3^). After stirring for 2.5 h at 80-90 °C, water (120 cm^3^) was added to the cooled reaction mixture and the resulting mixture was extracted with *tert*-butyl methyl ether (4 x 60 cm^3^). The combined extracts were dried (sodium sulfate), filtered and evaporated to give a yellow oil containing a white, crystalline solid. Recrystallisation of the residue from methanol gave the *title compound* **5b** (2.65 g, 71%) as a white, crystalline solid. (Found MNH_4_^+^ 252.1131, C_15_H_14_N_3_O requires 252.1131); m.p. 105-107 °C;* *_H_ (300 MHz; DMSO-d_6_) 3.73 (3H, s, C*H*_3_O), 7.67-7.79 (3H, complex, arylC*H*), 7.83 (1H, *ca* dd, *J* = 3.0 and 7.5, arylC*H*), 7.89 (1H, *ca* dd, *J* = 3.0 and 6.0, arylC*H*), 8.14 (1H, *ca* dd, *J* = 3.0 and 9.0, aryl C*H*) and 8.28 (1H, d, *J* = 9.0, arylC*H*);* *_C_ (75 MHz; DMSO-d_6_) 61.0 (*C*H_3_O), 67.8 (*C*(CN)_2_), 112.1 (*C*≡N), 113.4 (*C*≡N), 123.6, 125.4, 125.7, 127.6, 128.9(3), 128.9(5), 129.0, 129.2, 132.9(7), 132.9(9) (aryl*C*H and aryl*C*) and 186.19 (CH_3_O*C*).

**2-(1-Methoxy-2-(napthalen-1-yl)ethylidene)malononitrile 5c**

Crude 2-(1-hydroxy-2-(naphthalen-1-yl)ethylidene)malononitrile **4c** (4.26 g, assumed to be 18.2 mmol) and dimethyl sulfate (12.0 cm^3^, 126.8 mmol) were added to a stirred mixture of sodium hydrogen carbonate (12.23 g, 145.6 mmol), 1,4-dioxane (32 cm^3^) and water (5.4 cm^3^). After stirring for 2.5 h at 80-90 °C, water (80 cm^3^) was added to the cooled reaction mixture and the resulting mixture was extracted with *tert*-butyl methyl ether (3 x 70 cm^3^). The combined extracts were dried (sodium sulfate), filtered and evaporated to give a red/brown syrup, which was purified by flash chromatography on silica gel (66% hexane-34% ethyl acetate) to give two fractions. Fraction 1 was found to be methyl-2-(naphthalen-1-yl)acetate (1.335 g, 37%), obtained as an orange oil, with analytical data corresponding to literature values. Fraction 2 proved to be the *title compound* (423 mg, 9%), obtained as an orange/brown syrup which subsequently crystallised to an orange/brown solid. m.p. 110-112 °C;* *_H_ (300 MHz; CDCl_3_) 3.95 (3H, s, C*H*_3_O), 4.47 (2H, s, C*H*_2_), 7.28 (1H, *ca* dd, *J* = 0.5 and 6.0, arylC*H*), 7.52 (1H, *ca* t, *J* = 6.0 Hz, arylC*H*), 7.62 (2H, m, arylC*H*), 7.89 (1H, d, *J* = 9.0, arylC*H*) and 7.94 (2H, m, arylC*H*);* *_C_ (75 MHz; CDCl_3_) 34.3 (*C*H_2_), 59.4 (*C*H_3_O), 67.7 (*C*(CN)_2_), 111.5 (*C*≡N), 113.2 (*C*≡N), 122.2, 125.0, 125.5, 126.5, 127.2 (aryl*C*H), 127.6 (aryl*C*), 129.0, 129.2 (aryl*C*H), 130.9, 133.9 (aryl*C*) and 186.5 (CH_3_O*C*).

Construction of the fused heterocyclic core started with formation of 5-aminopyrazoles **6a**-**c** from enol ethers **5a**-**c** using *tert*-butylhydrazine.

**5-Amino-1-*tert*-butyl-3-(4-methylphenyl)-1*H*-pyrazole-4-carbonitrile 6a**

A mixture of 2-(methoxy(4-methylphenyl)methylene)malononitrile **5a** (792 mg, 4.0 mmol), *tert*-butylhydrazine hydrochloride (500 mg, 4.0 mmol) and triethylamine (0.56 cm^3^, 4.0 mmol) in ethanol (20 cm^3^) was heated under reflux for 3.5 h. The ethanol was evaporated *in vacuo*, water (30 cm^3^) was added and the resulting solid residue was filtered off and recrystallised from ethanol-water to give the *title compound* **6a** (716 mg, 71%) as fine, white needles. (Found MH^+^ 255.1605, C_15_H_19_N_4_ requires 255.1604); m.p. 164-166 °C;* *_H_ (300 MHz; DMSO-d_6_) 1.58 (9H, s, (C*H*_3_)_3_C), 2.34 (3H, s, C*H*_3_Ar), 6.35 (2H, broad s, N*H*_2_) and 7.27, 7.69 (2 x 2H, AA'BB', *J* = 9.0, arylC*H*);* *_H_ (75 MHz; DMSO-d_6_) 21.1 (*C*H_3_Ar), 28.4 ((*C*H_3_)_3_C), 59.3 ((CH_3_)_3_*C*), 72.3 (pyrazole *C*-4), 116.3 (*C*≡N), 125.7 (aryl*C*H), 129.3 (aryl*C*), 129.4 (aryl*C*H), 138.1 (aryl*C*CH_3_), 146.8 (pyrazole *C*-3) and 152.5 (pyrazole *C*-5).

**5-Amino-1-*tert*-butyl-3-(naphthalen-1-yl)-1*H*-pyrazole-4-carbonitrile 6b**

A mixture of 2-(methoxy(naphthalen-1-yl)methylene)malononitrile **5b** (2.20 g, 9.4 mmol), *tert*-butylhydrazine hydrochloride (1.17 g, 9.4 mmol) and triethylamine (1.31 cm^3^, 9.4 mmol) in ethanol (40 cm^3^) was heated under reflux for 3.5 h. The ethanol was evaporated *in vacuo*, the resulting residue was partitioned between water (50 cm^3^) and ethyl acetate (50 cm^3^) and the separated aqueous phase was further extracted with ethyl acetate (2 x 50 cm^3^). The combined organic extracts were dried (magnesium sulfate), filtered and evaporated to give an orange oil which was purified by flash chromatography on silica gel (50% hexane-50% ethyl acetate) to give the *title compound* **6b** (171.1 mg, 6%) as a pale yellow, crystalline solid.* *_H_ (300 MHz; CDCl_3_) 1.72 (9H, s, (C*H*_3_)_3_C), 4.49 (2H, broad s, N*H*_2_), 7.48-7.59 (3H, complex, arylC*H*), 7.74 (1H, *ca* dd, *J* = 2 and 6.0, arylC*H*), 7.86-7.92 (2H, complex, arylC*H*) and 8.46 (1H, m, arylC*H*);* *_C_ (75 MHz; CDCl_3_) 29.2 ((*C*H_3_)_3_C), 60.3 ((CH_3_)_3_*C*), 78.9 (pyrazole *C*-4), 115.0 (*C*≡N), 125.2, 125.9, 126.0, 126.5, 127.6, 128.3 (aryl*C*H), 128.6 (aryl*C*), 129.4 (aryl*C*H), 131.1 (aryl*C*), 134.0 (aryl*C*), 149.0 (pyrazole *C*-3) and 150.6 (pyrazole *C*-5).

**5-Amino-1-*tert*-butyl-3-(naphthalen-1-ylmethyl)-1*H*-pyrazole-4-carbonitrile 6c**

A mixture of 2-(1-methoxy-2-(napthalen-1-yl)ethylidene)malononitrile **5c** (382.1 mg, 1.54 mmol), *tert*-butylhydrazine hydrochloride (192.0 mg, 1.54 mmol) and triethylamine (0.22 cm^3^, 1.58 mmol) in ethanol (10 cm^3^) was heated under reflux for 3.5 h. The ethanol was evaporated *in vacuo*, the resulting residue was partitioned between water (25 cm^3^) and ethyl acetate (25 cm^3^) and the separated aqueous phase was further extracted with ethyl acetate (2 x 25 cm^3^). The combined organic extracts were washed with saturated aqueous sodium chloride solution (25 cm^3^), dried (magnesium sulfate), filtered and evaporated to give an orange syrup which was purified by flash chromatography on silica gel (50% hexane-50% ethyl acetate) to give the *title compound* **6c** (256.3 mg, 55%) as a pale orange, crystalline solid. * *_H_ (300 MHz; CDCl_3_) 1.60 (9H, s, (C*H*_3_)_3_C), 4.19 (2H, broad s, N*H*_2_), 4.35 (2H, s, C*H*_2_), 7.40-7.54 (4H, complex, arylC*H*), 7.77 (1H, *ca* d, *J* = 9.0, arylC*H*), 7.85 (1H, m, arylC*H*) and 8.30 (1H, m, arylC*H*);* *_C_ (75 MHz; CDCl_3_) 29.1 ((*C*H_3_)_3_C), 32.1 (*C*H_2_), 59.8 ((CH_3_)_3_*C*), 78.0 (pyrazole *C*-4), 114.5 (*C*≡N), 124.4, 125.5(0) (aryl*C*H), 125.5(2) (aryl*C*), 125.7, 127.4, 127.6, 128.5 (aryl*C*H), 132.1, 133.7, 133.9 (aryl*C*), 149.6 (pyrazole *C*-3) and 150.3 (pyrazole *C*-4).

Condensation of the 5-aminopyrazoles **6a**-**c** with formamide produced all three of the required inhibitors **1a**-**c** as crystalline solids, which were found to have analytical data consistent with those previously reported (Bishop et al., 1999).

**4-Amino-1-*tert*-butyl-3-(4-methylphenyl)-1*H*-pyrazolo[3,4-*d*]pyrimidine 1a (PP1)**

A solution of 5-amino-1-*tert*-butyl-3-(4-methylphenyl)-1*H*-pyrazole-4-carbonitrile **6a** (600 mg, 2.36 mmol) in formamide (7 cm^3^, 176 mmol) was heated under reflux for 3 h. Water (15 cm^3^) was added to the cooled solution and the brown-black solid produced was filtered off and dried *in vacuo*, before dissolution in hot ethanol (*ca* 20 cm^3^). The resulting solution was decolourised with activated charcoal, then filtered and the solvent evaporated *in vacuo* to give the title compound **1a** (456 mg, 69%) as an off-white, crystalline solid. * *_H_ (300 MHz; DMSO-d_6_) 1.76 (9H, s, (C*H*_3_)_3_C), 2.40 (3H, s, C*H*_3_Ar), 7.37, 7.56 (2 x 2H, AA'BB', *J* = 9, arylC*H*) and 8.25 (1H, s, pyrimidineC*H*) (Note: NH_2_ appeared as a very broad resonance between *ca* 5.0 and 6.5 ppm.);* *_C_ (75 MHz; DMSO-d_6_) 21.1 (*C*H_3_Ar), 29.0 ((*C*H_3_)_3_C), 59.8 ((CH_3_)_3_*C*), 98.9 (heterocyclic*C*), 128.5, 129.9 (aryl*C*H), 130.7, 138.1, 141.9, 154.0 (aryl*C*), 155.9 (pyrimidine*C*H) and 158.4 (aryl*C*H).

**4-Amino-1-*tert*-butyl-3-(naphthalen-1-yl)-1*H*-pyrazolo[3,4-*d*]pyrimidine 1b**

A solution of 5-amino-1-*tert*-butyl-3-(naphthalen-1-yl)-1*H*-pyrazole-4-carbonitrile **6b** (171.0 mg, 0.59 mmol) in formamide (2.7 cm^3^, 68.0 mmol) was heated under reflux for 3 h. Water (10 cm^3^) was added to the cooled solution and the brown-black solid produced was filtered off and dried *in vacuo*, before dissolution in hot ethanol (*ca* 12 cm^3^). The resulting solution was decolourised with activated charcoal, then filtered and the solvent evaporated *in vacuo* to give the title compound **1b** (71.1 mg, 38%) as a buff-coloured, crystalline solid. (Found MH^+^ 318.1717, C_19_H_20_N_5_ requires 318.1713); m.p. 211-214 °C;* *_H_ (300 MHz; DMSO-d_6_) 1.80 (9H, s, (C*H*_3_)_3_C), 7.51-7.71 (4H, complex, arylC*H*), 7.88 (1H, dd, *J* = 1.0 and 8.5, arylC*H*), 8.04-8.14 (2H, complex, arylC*H*) and 8.29 (1H, s, pyrimidineC*H*) [lit.^2^ *δ*_H_ (270 MHz; CDCl_3_) 1.92 (9H, s), 5.04 (2H, m), 7.43-7.73 (4H, m), 7.92-8.02 (3H, m) and 8.34 (1H, s)];* *_C_ (75 MHz; DMSO-d_6_) 29.1 ((*C*H_3_)_3_C), 60.0 ((CH_3_)_3_*C*), 100.7 (heterocyclic*C*) and 125.6, 125.9, 126.5, 127.1, 128.5, 128.6, 129.4, 130.4, 131.7, 133.4, 140.2, 153.7, 155.1, 158.2 (aryl*C*H, aryl*C*, heterocyclic*C*H and heterocyclic*C*).

**4-Amino-1-*tert*-butyl-3-(naphthalen-1-ylmethyl)-1*H*-pyrazolo[3,4-*d*]pyrimidine 1c**

A solution of 5-amino-1-*tert*-butyl-3-(naphthalen-1-ylmethyl)-1*H*-pyrazole-4-carbonitrile **6c** (256.3 mg, 0.84 mmol) in formamide (3.8 cm^3^, 95.6 mmol) was heated under reflux for 3 h. Water (15 cm^3^) was added to the cooled solution and the brown-black solid produced was filtered off and dried *in vacuo*, before dissolution in hot ethanol (*ca* 10 cm^3^). The resulting solution was decolourised with activated charcoal, then filtered and the solvent evaporated *in vacuo* to give the title compound **1c** (181.3 mg, 65%) as a buff-coloured, crystalline solid. (Found MH^+^ 332.1872, C_20_H_22_N_5_ requires 332.1870); m.p. 172-175 °C;* *_H_ (300 MHz; DMSO-d_6_) 1.68 (9H, s, (C*H*_3_)_3_C), 4.80 (2H, s, C*H*­_2_), 7.01 (2H, broad s, N*H*_2_), 7.16 (1H, d, *J* = 7.0, arylC*H*), 7.41 (1H, t, *J* = 7.1, arylC*H*), 7.56 (2H, m, arylC*H*), 7.81 (1H, d, *J* = 8.2, arylC*H*), 7.94 (1H, m, arylC*H*), 8.15 (1H, s, pyrimidineC*H*) and 8.34 (1H, m, arylC*H*) [lit.^2^ *δ*_H_ (270 MHz; CDCl_3_) 1.85 (9H, s), 4.76 (2H, s), 5.04 (2H, s), 7.19 (1H, d, *J* = 6), 7.39 (1H, t, *J* = 8), 7.55 (2H, t, *J* = 4), 7.79-7.92 (2H, m), 8.20 (1H, d, *J* = 8) and 8.24 (1H, s)];* *_C_ (75 MHz; DMSO-d_6_) 29.0 ((*C*H_3_)_3_C), 31.4 (*C*H_2_), 59.4 ((CH_3_)_3_*C*), 100.7 (heterocyclic*C*) and 124.6, 125.7, 125.8, 126.0, 126.1, 127.2, 128.6, 131.3, 133.6, 135.4, 140.6, 154.1, 154.8, 158.4, (aryl*C*H, aryl*C*, heterocyclic*C*H and heterocyclic*C*).

**References**

Bishop, A. C., Kung, C. Y., Shah, K., Witucki, L., Shokat, K. M. & Liu, Y., (1999) Generation of monospecific nanomolar tyrosine kinase inhibitors via a chemical genetic approach. *J. Am. Chem. Soc.* **121**: 627-631.

Bishop, A. C., Ubersax, J. A., Petsch, D. T., Matheos, D. P., Gray, N. S., Blethrow, J., Shimizu, E., Tsien, J. Z., Schultz, P. G., Rose, M. D., Wood, J. L., Morgan, D. O. & Shokat, K. M., (2000) A chemical switch for inhibitor-sensitive alleles of any protein kinase. *Nature* **407**: 395-401.

Hanefeld, U., Rees, C. W., White, A. J. P. & Williams, D. J., (1996) One-pot synthesis of tetrasubstited pyrazoles - Proof of regiochemistry. *J. Chem. Soc., Perkin Trans. 1*: 1545-1552.

Wood, M. E., Bissiriou, S., Lowe, C., Norrish, A. M., Sénéchal, K., Windeatt, K. M., Coles, S. J. & Hursthouse, M. B., (2010) Synthetic use of the primary kinetic isotope effect in hydrogen atom transfer: generation of a-aminoalkyl radicals. *Org. Biomol. Chem.* **8**: 4653.

**Acknowledgements**

We thank the EPSRC National Mass Spectrometry Service Centre (Swansea, UK) for determination of mass spectra.
