# Supplementary material for: Protein kinase C is essential for viability of the rice blast fungus M agnaporthe oryzae
Source: Mol Microbiol. 2015 Aug 18;98(3):403–19. doi: 10.1111/mmi.13132 (PMC4791171; doi:10.1111/mmi.13132)
Supplement: Supplementary file 1 — Supporting information [file MMI-98-403-s001.zip › MMI_13132_supp-0009-Table_S1.docx]

| Sample | Replicate | Total Mapped Reads |
| --- | --- | --- |
| T0 | 1 | 9973306 |
| T0 | 2 | 26312816 |
| T1_POS | 1 | 22394974 |
| T1_POS | 2 | 1894449 |
| T3_POS | 1 | 23340446 |
| T3_POS | 2 | 31638424 |
| T6_POS | 1 | 30279587 |
| T6_POS | 2 | 14961651 |
| T12_POS | 1 | 26733026 |
| T12_POS | 2 | 16477417 |
| T24_POS | 1 | 26337243 |
| T24_POS | 2 | 20966330 |
| T1_NEG | 1 | 21103946 |
| T1_NEG | 2 | 24547545 |
| T3_NEG | 1 | 35089560 |
| T3_NEG | 2 | 20814173 |
| T6_NEG | 1 | 20219922 |
| T6_NEG | 2 | 28971043 |
| T12_NEG | 1 | 20122894 |
| T12_NEG | 2 | 21970339 |
| T24_NEG | 1 | 28228317 |
| T24_NEG | 2 | 34761469 |

**Table S1. Total mapped reads following RNA-Seq analysis**

Total number of RNA-seq reads that mapped to the reference genome for each sample. T= number of hours after start of experiment, POS = PP1 inhibitor added, NEG = untreated control.

Library problem
