# Supplementary figures and images for: Protein kinase C is essential for viability of the rice blast fungus M agnaporthe oryzae
Source: Mol Microbiol. 2015 Aug 18;98(3):403–19. doi: 10.1111/mmi.13132 (PMC4791171; doi:10.1111/mmi.13132)

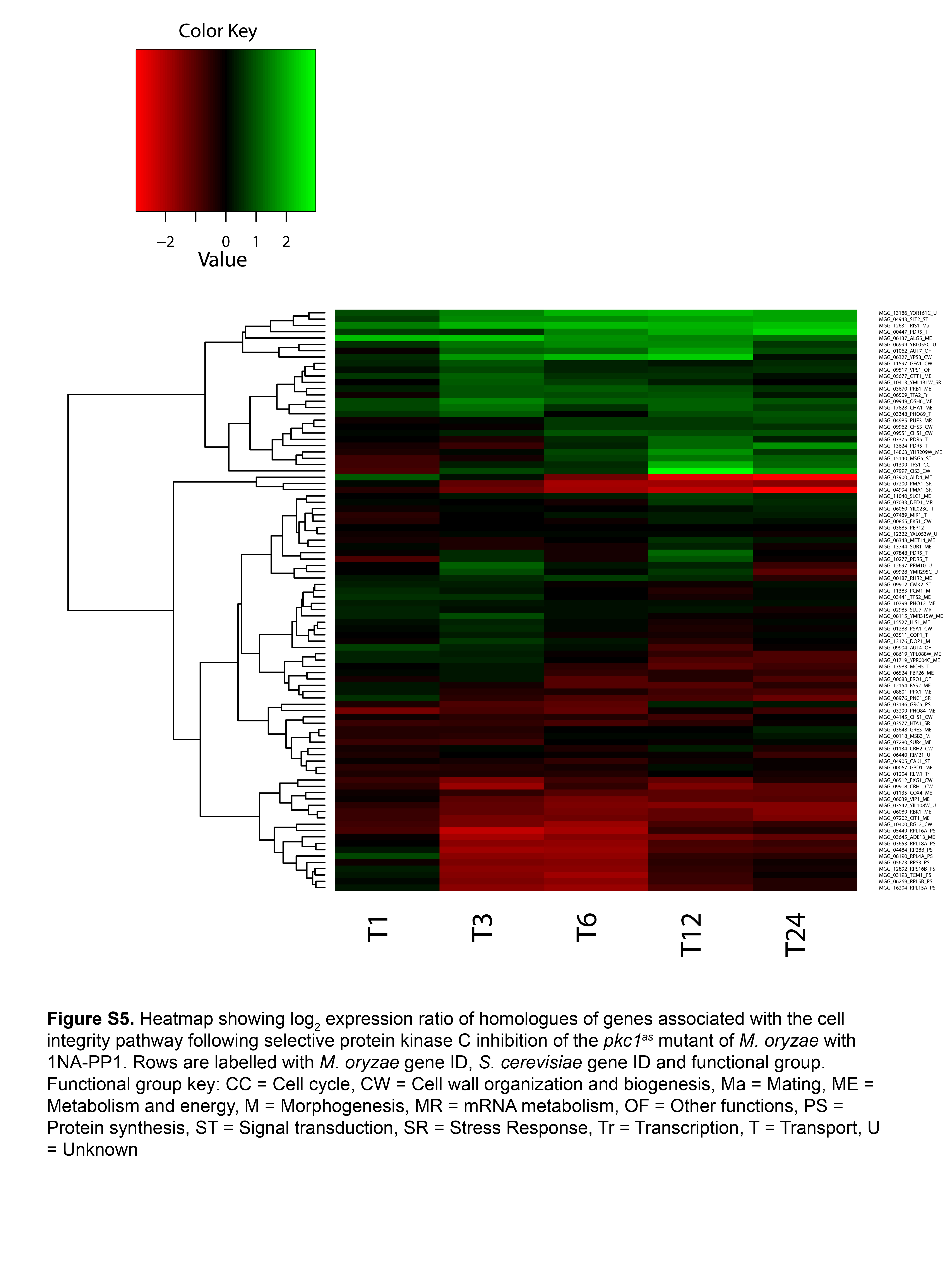

Supplement: Supplementary file 1 — Supporting information [file MMI-98-403-s001.zip › MMI_13132_supp-0005-Figure_S5_new.tif]

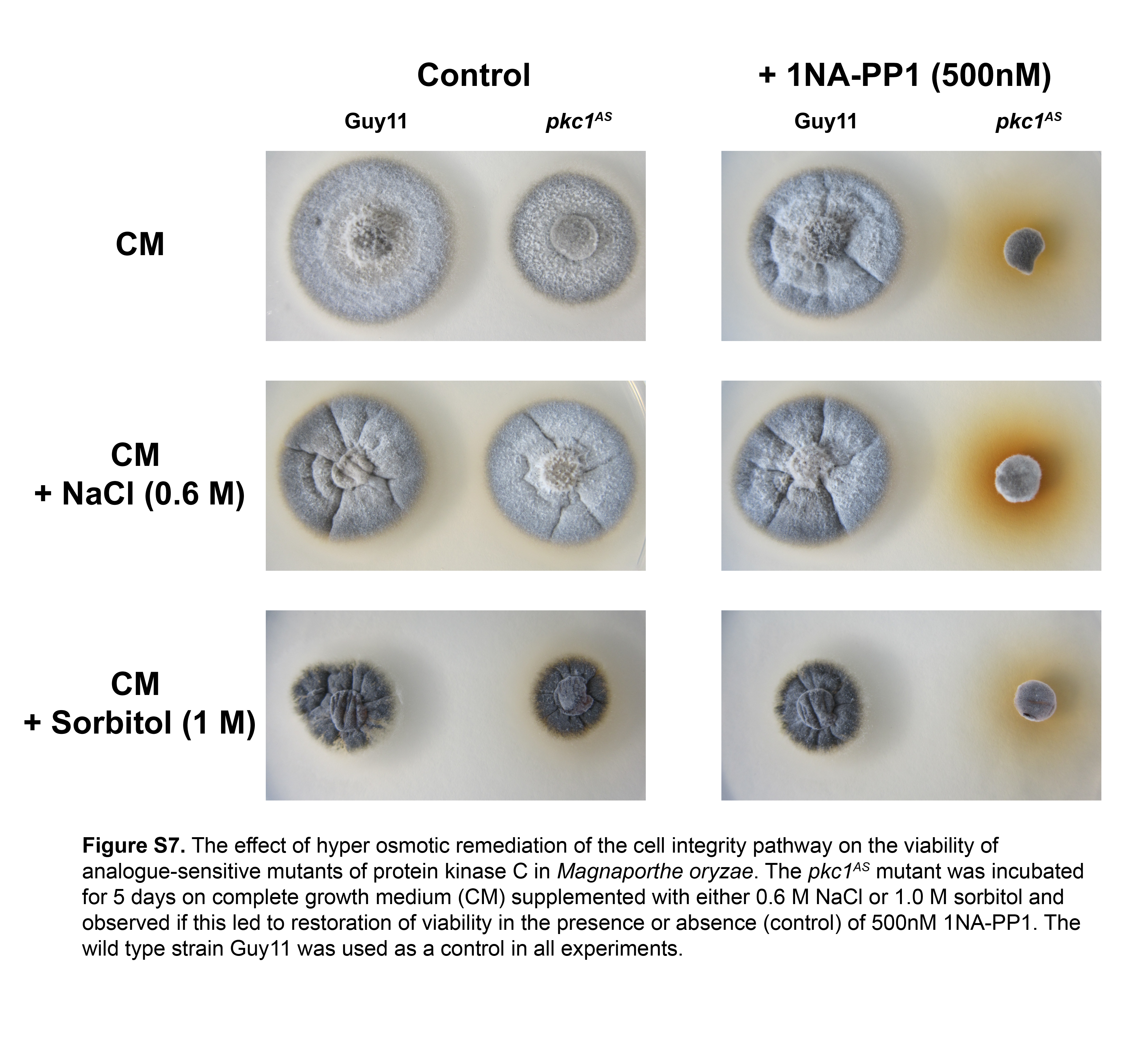

Supplement: Supplementary file 1 — Supporting information [file MMI-98-403-s001.zip › MMI_13132_supp-0007-Figure_S7_%28osm%29_new.tif]
